# Amino acid reprogramming and biofilm-specific tricarboxylate transporters in PET-degrading *Piscinibacter sakaiensis*

**DOI:** 10.1128/aem.01465-26

**Published:** 2026-09-08

**Authors:** Hao Wei Lim, Jiabao Xu, Jonas Koh, Bin Cao, Weiming Tu

**Affiliations:** 1 School of Civil and Environmental Engineering, Nanyang Technological University 54761, , Singapore, Singapore; 2 Singapore Centre for Environmental Life Sciences Engineering, Nanyang Technological University 54761, , Singapore, Singapore; 3 James Watt School of Engineering, University of Glasgow 3526, , Glasgow, United Kingdom; 4 The Gene and Linda Voiland School of Chemical Engineering and Bioengineering, Washington State University 6760, Pullman, Washington, USA; 5 Nanyang Environment and Water Research Institute, Nanyang Technological University 54761, Singapore, Singapore; University of Milano-Bicocca, Milan, Italy

**Keywords:** PET biodegradation, biofilm metabolism, bacterial transcriptomics, single-cell Raman spectroscopy, tripartite tricarboxylate transporter, terephthalate

## Abstract

Plastic-degrading bacteria predominantly colonize polymer surfaces as biofilms, yet it remains unclear whether the biofilm phenotype contributes to metabolism beyond retaining extracellular enzymes. Here, we combine population-level RNA-sequencing across three conditions—biofilm cells on polyethylene terephthalate (PET), planktonic cells incubated with PET, and planktonic cells on maltose—with single-cell Raman spectroscopy to characterize the PET response of *Piscinibacter sakaiensis* (formerly *Ideonella sakaiensis*). This integrated approach reveals two metabolically distinct response layers. A carbon-source-driven response shared by all PET-exposed cells is dominated by a broad amino acid reprogramming, led by upregulation of branched-chain amino acid transport genes, enhanced serine biosynthesis, and reduced chemotaxis. A biofilm-specific layer selectively induces tripartite tricarboxylate transporter genes from three distinct genomic loci. This transcriptional feature is accompanied by a single-cell phenotype consistent with a protein-rich and saturated membrane. These results suggest that biofilm formation is not limited to enzyme retention but is associated with selective activation of transport systems, consistent with a putative role in capturing PET-derived intermediates at the polymer interface. This two-layer model separates general metabolic adaptation to PET from biofilm-specific functions and provides a framework for understanding how surface-associated bacterial physiology contributes to plastic degradation.

**IMPORTANCE:**

Polyethylene terephthalate (PET) degradation in natural and engineered environments is largely mediated by surface-attached microbial communities, yet the physiological role of biofilm state during plastic degradation remains poorly understood. Using the model PET degrader *Piscinibacter sakaiensis*, we show that biofilm-associated cells are not simply retained near the polymer surface but exhibit a distinct metabolic program characterized by selective induction of tripartite tricarboxylate transporters. In contrast, extensive amino acid reprogramming occurs in both biofilm and planktonic PET-exposed cells, indicating that it is driven by carbon source rather than surface attachment. These findings reveal that PET degradation involves two separable physiological layers: a general metabolic response to PET-derived carbon shared across cell phenotypes, and a biofilm-specific transport response potentially linked to substrate capture at the plastic interface. This work advances our understanding of how microbial physiology is organized during plastic biodegradation and identifies transport processes as previously unrecognized components of PET-degrading biofilms.

## INTRODUCTION

Plastic accumulation in the environment is a planetary-scale waste-management problem (1–3), and biological recycling is one of the few credible routes to closing the carbon loop rather than relocating the waste (4, 5). Global plastic production has exceeded 8,300 million tons, of which roughly 80% has accumulated in landfills or the environment (6). Current annual aquatic emissions, estimated at 19–23 million tons, are projected to increase to 53 million tons per year by 2030 in the absence of effective intervention (7). Among synthetic polymers, polyethylene terephthalate (PET), the most-produced thermoplastic polyester and the dominant material in single-use beverage bottles and textile fibers, has emerged as the primary target for biological depolymerization. Over the past decade, substantial progress has been made in identifying PET-degrading microorganisms and engineered PET-hydrolyzing enzymes including cutinases and PETase variants (8).

To date, engineering efforts have concentrated on the secreted hydrolases that initiate PET breakdown. Variants of leaf-branch compost cutinase achieve over 90% PET depolymerization in 10 h under industrial conditions (9), while machine-learning-guided PETase variants extend catalytic activity to ambient temperatures (10). The downstream enzyme MHETase has also been characterized at the structural and mechanistic levels (11). In parallel, metagenomic mining of plastisphere communities, including activated-sludge, marine, and landfill metagenomes, has expanded the catalog of candidate PET-active esterases, with hundreds of putative PETase homologs now reported (12–14). Biofilm formation is commonly invoked to explain enhanced degradation, with the prevailing view that the biofilm matrix functions as an extracellular digestive system that retains secreted enzymes near the polymer surface (15). However, the metabolic activities of cells within the biofilm itself remain poorly understood, and the extent to which the biofilm phenotype reorganizes intracellular metabolism during PET degradation is unresolved.

*Piscinibacter sakaiensis* 201-F6, originally described as *Ideonella sakaiensis* 201-F6 (16, 17), remains the best-characterized bacterium with a dedicated PET-degradation pathway. PETase cleaves PET to the soluble mono(2-hydroxyethyl) terephthalate (MHET), which is subsequently hydrolyzed by MHETase to terephthalate (TPA) and ethylene glycol (EG). Downstream EG catabolism through dedicated periplasmic and cytoplasmic dehydrogenases (18), as well as proteome-level induction of MHETase, the tricarboxylic acid (TCA) cycle, and β-oxidation under PET, TPA, and EG growth (19), has been documented in planktonic culture. In contrast, to our knowledge, the transcriptomic response of *P. sakaiensis* growing as a biofilm on PET, the form in which environmental polymer degradation actually occurs, has not been systematically characterized. Understanding biofilm–plastic interactions is therefore essential for elucidating how bacterial metabolism is reorganized during PET degradation and how biofilm-associated physiology contributes to polymer turnover.

**Fig 1:**
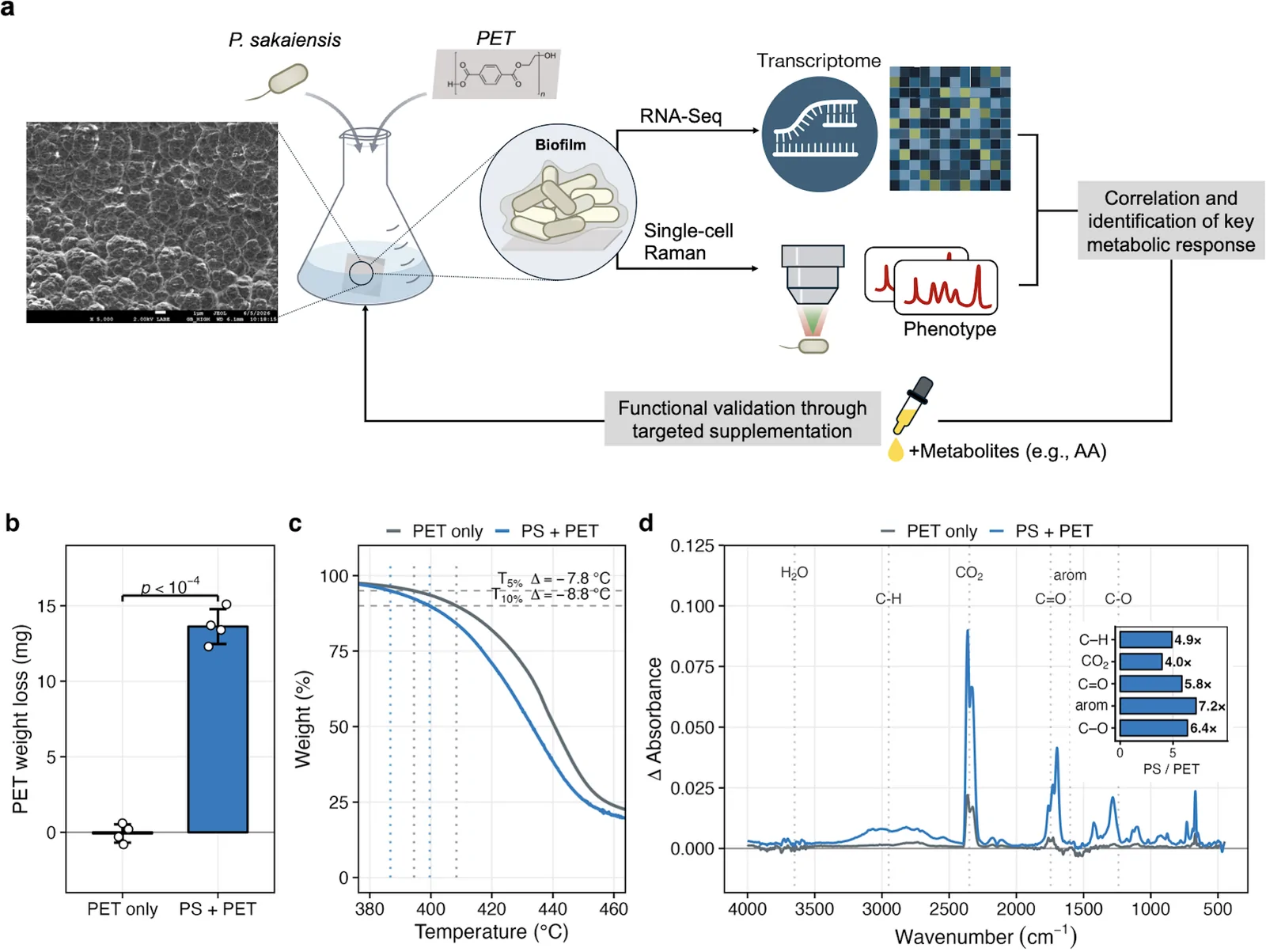
*Piscinibacter sakaiensis* degrades PET films. (**a**) Experimental workflow. *P. sakaiensis* biofilms cultured on amorphous PET films (1.9% crystallinity) for 32 days at 30°C without shaking in chemically defined SV medium with PET as the sole carbon source (left: scanning electron micrograph of the biofilm surface, scale bar 10 µm) are profiled in parallel by bulk RNA-seq (population transcriptome) and single-cell Raman spectroscopy (single-cell phenotype). Correlated transcriptome–phenotype hits are functionally validated by targeted metabolite supplementation (e.g., amino acids). (**b**) PET film mass loss after 32 days of incubation with *P. sakaiensis* (PS + PET) vs a sterile no-bacteria control (PET only). Bars show group means; error bars represent ±1 SD (*n* = 4 films per group); and the *P* value was determined by a two-sided Welch’s *t*-test. (**c**) Thermogravimetric analysis of films recovered at the end of the culture window, zoomed to the 380°C–460°C polymer-decomposition region. Dashed lines mark 95% and 90% residual mass at *T*_5_% and *T*_10_% onset temperatures of each sample. PS + PET decomposes 7.8°C earlier at *T*_5_% and 8.8°C earlier at *T*_10_% than virgin PET. (**d**) TG-FTIR difference spectra (Δ absorbance vs baseline) of evolved gases captured at peak gas-evolution temperatures. IR bands are labeled at H_2_O (3,650 cm^−1^), aliphatic C–H (2,950 cm^−1^), CO_2_ (2,350 cm^−1^), ester C = O (1,745 cm^−1^), aromatic ring (1,600 cm^−1^), and C–O (1,240 cm^−1^). (Inset) Per-band absorbance fold change (PS + PET/PET only)**.**

Here, we investigated the metabolic and physiological basis of PET-associated biofilm growth using an integrated experimental design (Fig. 1a). The same set of *P. sakaiensis* cultures, grown statically on amorphous PET films in a chemically defined medium for more than 4 weeks, was profiled by population-level RNA sequencing across three conditions (biofilm and planktonic cells on PET and planktonic cells on maltose) and three contrasts. These analyses were complemented by single-cell Raman spectroscopy of biofilm and planktonic cells harvested from the same cultures (20–22), together with amino acid supplementation experiments coupled to readout by confocal live/dead imaging and biofilm-resolved single-cell Raman analysis. The three-way transcriptomic design separates the PET response shared by biofilm and planktonic cells from the biofilm-specific layer above it, and the single-cell Raman provides an independent functional readout per cell. The PET response decomposes into a broad amino acid reprogramming shared across all PET-exposed cells and a sharply selective biofilm-specific layer on top, a decomposition the matrix-retention model alone does not predict.

## RESULTS

### *Piscinibacter sakaiensis* degrades PET films

To dissect how *P. sakaiensis* metabolizes PET, the wild-type strain was inoculated into a defined sodium carbonate–vitamin (SV) medium (a yeast-extract-free modification of the YSV medium of Yoshida et al. [16]) containing amorphous PET as the sole carbon source, enabling biofilm formation on the polymer surface (Fig. 1a). A maltose-grown planktonic culture served as a carbon-source control. After 32 days of incubation, PET films exposed to *P. sakaiensis* show measurable changes in both film mass and thermal stability. Bacteria-exposed films lost 13.6 ± 1.2 mg of starting material over the culture period, against −0.1 ± 0.6 mg for no-bacteria controls (*n* = 4 films per group; two-sided Welch’s *t*-test, *P* = 1 × 10^−5^, Cohen’s *d* = 14.9; Fig. 1b). Thermogravimetric analysis of the recovered films revealed an accompanying reduction in thermal stability across the polymer-decomposition window of 380°C–460°C (Fig. 1c). The temperature at which 5% of the original mass had been lost (*T*_5_%) occurred 7.8°C earlier in PS + PET than in virgin PET, and the *T*_10_% onset was 8.8°C earlier. A lower decomposition onset without a change in overall thermogram shape is the signature expected if surface polymer chains have been pre-shortened by extracellular enzymatic activity, rather than the bulk polymer being chemically modified.

To identify the chemical nature of degradation products, thermogravimetry coupled with evolved-gas Fourier-transform infrared spectroscopy (TG-FTIR) was performed; the spectrum recorded at each sample’s peak gas-evolution temperature provided a chemical fingerprint of the species released during decomposition. Bacteria-exposed films enriched every major pyrolysis product four- to sevenfold relative to virgin PET, with the aromatic-ring band at 1,600 cm^−1^ (7.2-fold) and the C–O band at 1,240 cm^−1^ (6.4-fold) showing the largest gains (Fig. 1d). The Gram–Schmidt root-mean-square trace of total evolved-gas absorbance confirms that this enrichment spans the full 380°C–500°C decomposition window rather than occurring at a single temperature (Fig. S1). Scanning electron microscopy of the colonized surface (Fig. 1a) and the side-by-side comparison with a smooth no-bacteria control at matched magnification (Fig. S2) confirm continuous biofilm formation. The gravimetric, thermal, chemical, and morphological evidence together demonstrates that *P. sakaiensis* materially erodes PET under these conditions.

### Single-cell Raman confirms active PET metabolism

**Fig 2:**
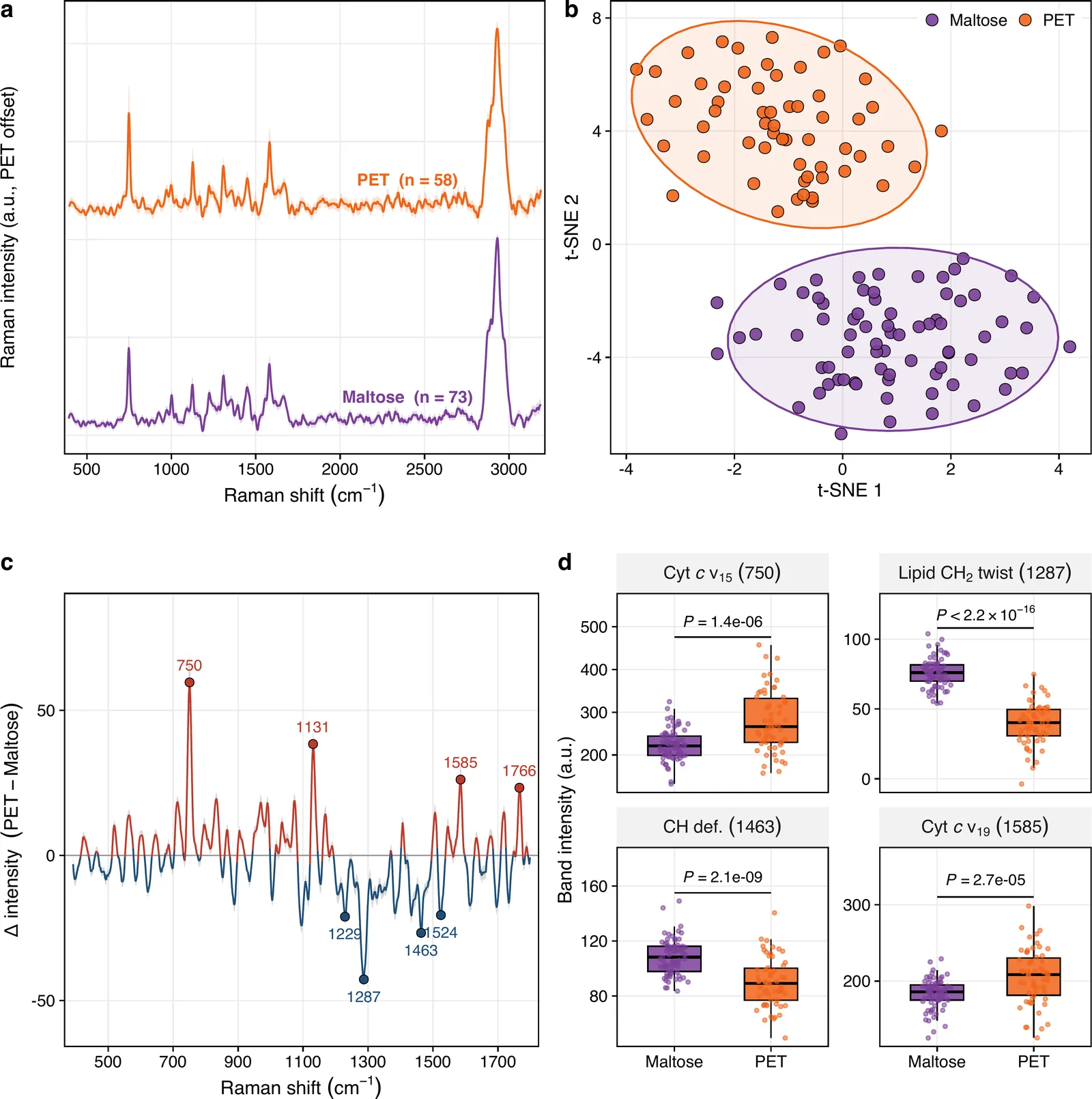
Single-cell Raman spectroscopy confirms active PET metabolism in *P. sakaiensis.* (**a**) Mean Raman spectra of *P. sakaiensis* cells harvested from planktonic cultures grown on maltose (*n* = 73 single-cell spectra) or on PET as the sole carbon source (*n* = 58), offset vertically for clarity. (**b**) t-Distributed stochastic neighbor embedding (t-SNE, perplexity = 30) of the same 131 spectra; shaded regions are 90% confidence ellipses. (**c**) Mean difference spectrum (PET − maltose) with biomarker peaks annotated; positive peaks (red) indicate bands enriched in PET-grown cells, and negative peaks (blue) indicate bands enriched in maltose-grown cells. (**d**) Band intensities at four biomarker wavenumbers: cytochrome c ν_15_ (750 cm^−1^), lipid CH_2_ twist (1,287 cm^−1^), generic CH deformation (1,463 cm^−1^), and cytochrome c ν_19_ (1,585 cm^−1^). Box plots show median and interquartile range; whiskers extend to 1.5 × IQR; individual spectra are overlaid as points. *q* values from two-sided Wilcoxon rank-sum tests (Benjamini–Hochberg corrected) comparing PET vs maltose groups.

Having established that *P. sakaiensis* materially degrades PET, we next asked whether cells on the polymer are metabolically active or instead colonizing the surface passively. To address this, we acquired single-cell Raman spectra from planktonic *P. sakaiensis* cells harvested from PET cultures (*n* = 58 spectra) and, as the reference carbon source, from cells grown on maltose (*n* = 73 spectra). Mean spectra from the two conditions differed visibly across the 600–1,800 cm^−1^ fingerprint region (Fig. 2a), and *t*-distributed stochastic neighbor embedding (t-SNE) of the same 131 spectra resolved maltose- and PET-grown cells into two non-overlapping clusters whose 90% confidence ellipses do not intersect (Fig. 2b).

To identify the molecular drivers of this separation, we computed the mean PET−maltose difference spectrum (Fig. 2c) and quantified four biomarker bands per spectrum (Fig. 2d). Cytochrome c bands at 750 cm^−1^ (ν_15_, pyrrole breathing) and 1,585 cm^−1^ (ν_19_, C_m_ methine), two independent markers of an active electron-transport chain (23), were both elevated in PET-grown cells (Cliff’s *δ* = +0.49 and +0.43; Fig. 2d). In parallel, the lipid CH_2_ twist at 1,287 cm^−1^ was depleted to near-complete group separation (Cliff’s *δ* = −0.96), and the generic CH deformation at 1,463 cm^−1^ was also strongly depleted (*δ* = −0.61), indicating concurrent remodeling of membrane acyl chains and bulk aliphatic content (24). All four contrasts are highly significant under two-sided Wilcoxon rank-sum tests (*q* ≤ 3 × 10^−5^). Elevated cytochrome c signals indicate active respiration in PET-associated cells rather than passive colonization, while concurrent membrane signatures preview the biofilm-specific phenotype quantified in “Biofilm cells exhibit a single-cell membrane-composition phenotype,” below.

### PET drives a coordinated, shared catabolic and lifestyle program

**Fig 3:**
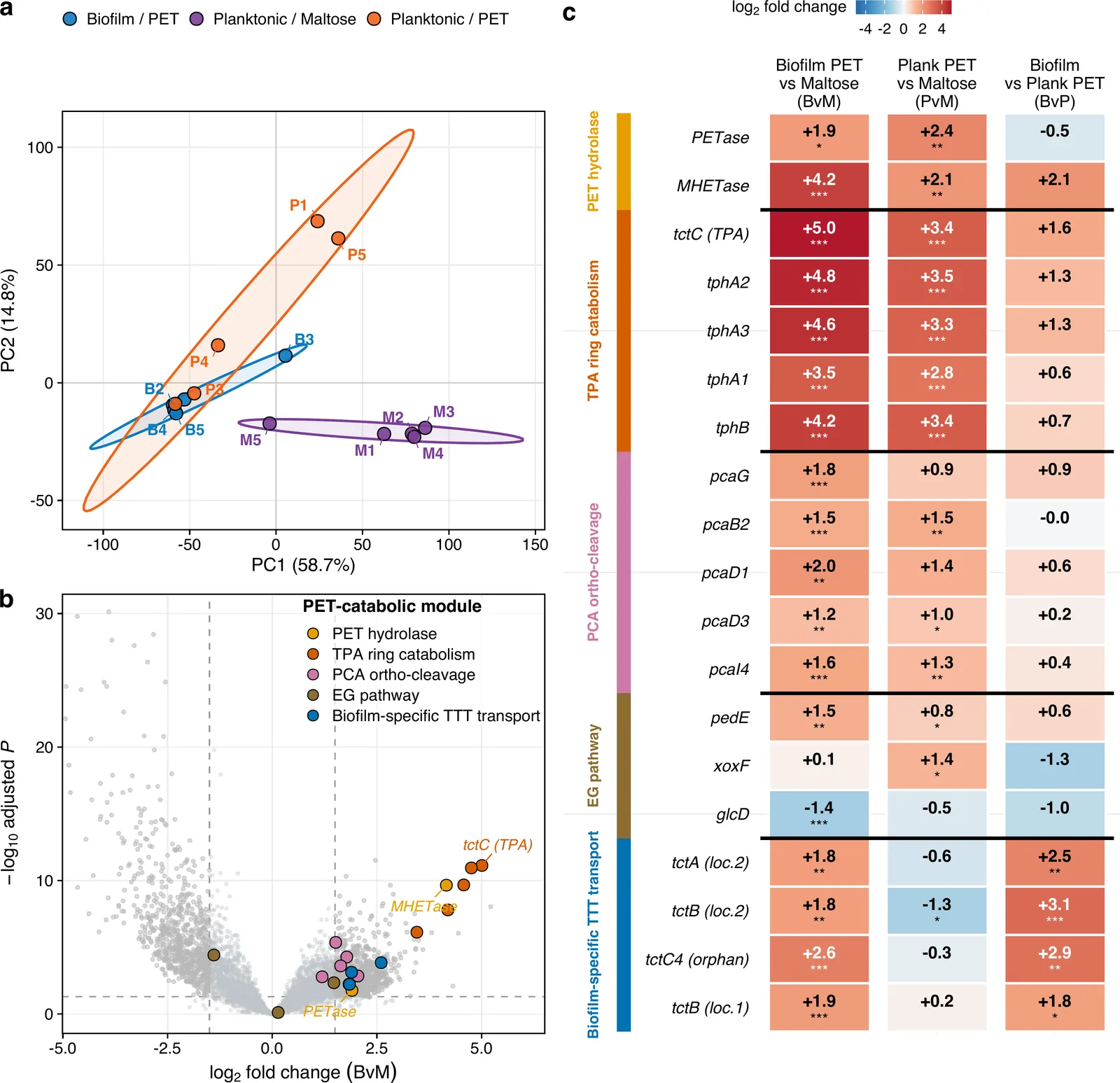
PET drives a coordinated, shared catabolic program in *P. sakaiensis***.** (**a**) Principal component analysis (PCA) of log_2_-normalized DESeq2 counts for all 15 RNA-seq libraries: biofilm cells on PET (*n* = 5), planktonic cells on PET (*n* = 5), and planktonic cells on maltose (*n* = 5). Shaded regions are 80% confidence ellipses; axes give the variance explained by each component. (**b**) Volcano plot of the biofilm-vs-maltose (BvM) contrast with the PET-catabolic pathway genes overlaid and colored by module (PET hydrolase, TPA-ring catabolism, protocatechuate [PCA] *ortho*-cleavage, EG pathway, and the biofilm-specific TTT transporters); PETase, MHETase, and the *tctC* (TPA) substrate-binding protein are labeled, and all other genes are gray. (**c**) Log_2_ fold-change heatmap of the 19 pathway and transporter genes across the three contrasts (BvM; planktonic-PET-vs-maltose, PvM; and biofilm-vs-planktonic-on-PET, BvP), grouped by module. Cell values are signed log_2_FC; asterisks denote significance after Benjamini–Hochberg correction (*p* adj <0.05, <0.01, and <0.001 shown as one, two, and three asterisks, respectively). The hydrolase, TPA-ring, and protocatechuate modules are induced in both BvM and PvM (the shared PET response), whereas the TTT transporters are induced only in biofilm cells (positive in BvM and BvP, flat or negative in PvM). Abbreviations: DEG, differentially expressed gene; EG, ethylene glycol; FC, fold change; PCA, protocatechuate (c) or principal component analysis (a); PET, polyethylene terephthalate; TPA, terephthalate; TTT, tripartite tricarboxylate transporter.

To examine the molecular basis of this metabolic activity, we performed RNA-seq on three culture conditions in five biological replicates each: biofilm cells on PET, planktonic cells on PET, and planktonic cells on maltose (15 libraries in total). Principal component analysis (PCA) on log_2_-normalized counts resolved the three conditions into separate clusters along PC1 (58.7% of variance) and PC2 (14.8%) (Fig. 3a). Sample-to-sample Spearman correlations confirmed replicate coherence within each condition (Fig. S3). Differential expression was called with DESeq2 ( *P* adj<0.05 and |log_2_FC| >1.5) for three contrasts: biofilm-vs-maltose (BvM), planktonic-PET-vs-maltose (PvM), and biofilm-vs-planktonic-on-PET (BvP).

We then traced the complete PET-catabolic route gene by gene across the three contrasts. Mapping the genes encoding PETase and MHETase, the TPA ring-cleavage operon (the *tphA1*–*A3* and *tphB* dioxygenase genes with their cognate substrate-binding protein), the protocatechuate *ortho*-cleavage branch, and the EG oxidation genes onto the BvM volcano, we found that the entire pathway fell within the upregulated half under PET growth (Fig. 3b). The same modules are induced in the PvM contrast (Fig. S4), so this catabolic program is a shared carbon-source response rather than a biofilm-specific one. The three-contrast pathway heatmap (Fig. 3c) resolves two tiers: the hydrolase, TPA-ring, and protocatechuate modules are strongly induced in both BvM and PvM but flat in BvP, whereas a small set of tripartite tricarboxylate transporter (TTT) genes is induced specifically in biofilm cells (positive in BvM and BvP, flat or negative in PvM; see “Biofilm cells specifically upregulate tripartite tricarboxylate transporters from three distinct genomic loci,” below). Full per-gene statistics for the 19 pathway and transporter genes are given in Table S1.

**Fig 4:**
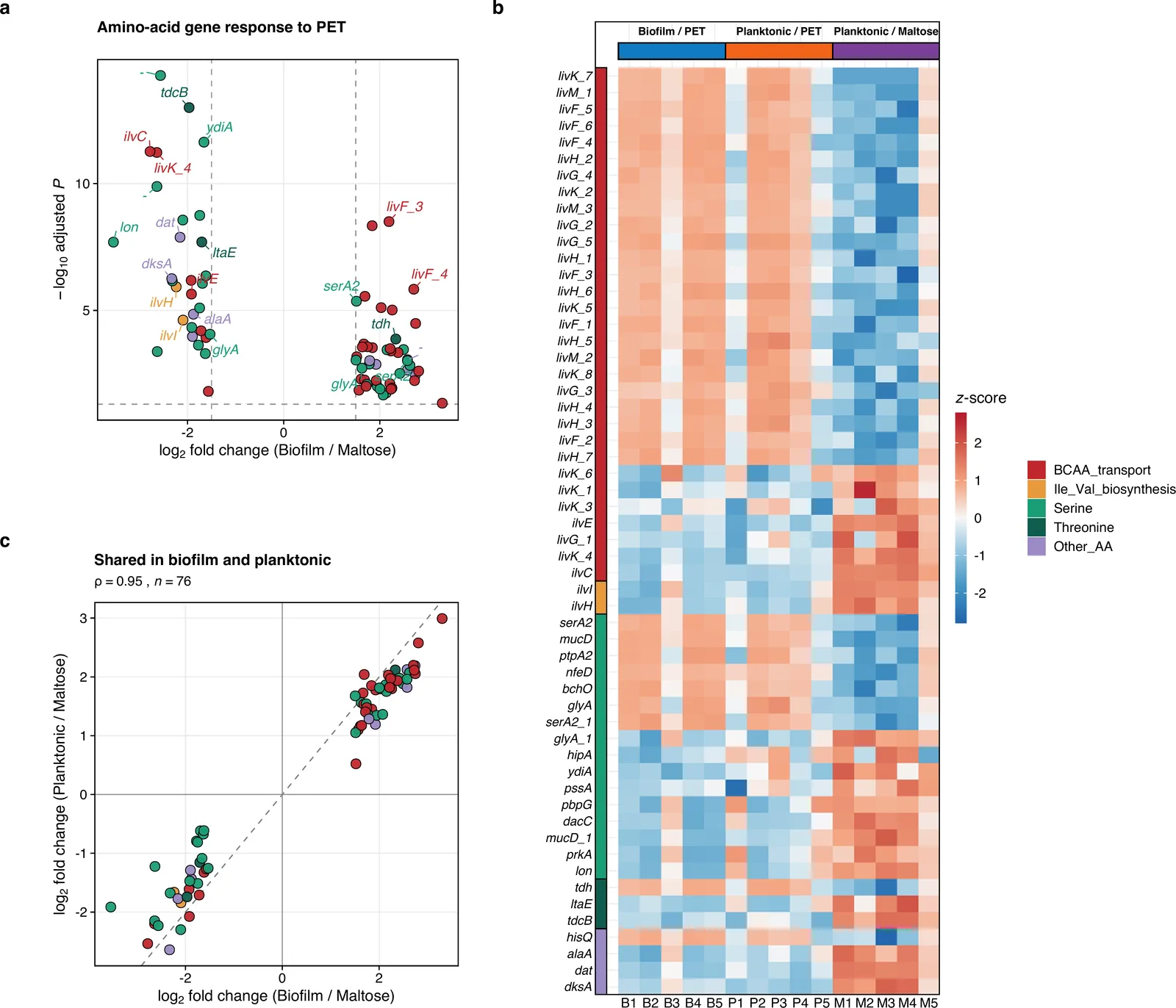
Branched-chain amino acid transport and serine biosynthesis drive the PET amino acid response, shared by biofilm and planktonic cells. (**a**) Volcano plot restricted to amino acid genes, plotted against the biofilm-vs-maltose (BvM) log_2_ fold change. Colors indicate AA subcategory: BCAA transport (red), Ile/Val biosynthesis (orange), serine (teal), threonine (dark teal), and other AA (lavender). Vertical dashed lines mark the |log_2_FC| = 1.5 threshold; the horizontal dashed line marks *P* adj = 0.05 (DESeq2 Wald test, Benjamini–Hochberg correction). (**b**) Per-sample expression heatmap of the 56 named significant AA DEGs across all 15 RNA-seq libraries, rows ordered by AA subcategory then by BvM log_2_FC, columns grouped by condition (biofilm/PET B1–B5, planktonic/PET P1–P5, and planktonic/maltose M1–M5). (**c**) Cross-contrast scatter of log_2_FC in BvM (*x*-axis) against log_2_FC in planktonic-vs-maltose (*y*-axis) for the same AA gene set as panel a; points colored by AA subcategory; dashed reference line marks *y* = *x*. Spearman *ρ* on log_2_FC and gene count annotated above the panel.

This coordinated induction extends beyond the catabolic pathway to a genome-wide lifestyle program that is likewise shared between biofilm and planktonic PET cells: flagellar and chemotaxis genes and the TCA cycle are broadly downregulated, while exopolysaccharide- and matrix-biosynthesis genes and membrane-lipid-remodeling genes—including cyclopropane-fatty-acid synthase (*cfa*) and lysyl-phosphatidylglycerol synthases—are upregulated (Fig. S5). Full statistics for the 139 genes in these modules are provided as Table S2. Amino acid transport and metabolism is the most prominent upregulated module within this shared layer; its branched-chain-transport and serine (Ser)-biosynthesis drivers are dissected next (Fig. 4).

### Branched-chain amino acid transport and serine biosynthesis drive the AA response

Restricting the BvM contrast to genes assigned an AA-category label, we found that the AA-only volcano (Fig. 4a) is dominated in the upregulated half by BCAA-transporter paralogs and serine-biosynthesis genes, while the first committed step of BCAA catabolism is suppressed. In particular, *ilvE* (branched-chain aminotransferase) is downregulated in both BvM (log_2_FC −1.92) and PvM (−2.07), so PET-exposed cells, biofilm or planktonic, import branched-chain amino acids without catabolizing them.

The per-sample expression heatmap of the 56 significant AA DEGs across all 15 RNA-seq libraries (Fig. 4b) reveals paralog-specific regulation within an expanded transporter family. *P. sakaiensis* encodes 29 *livKHMGF*-type ABC-transporter paralogs distributed across the chromosome, of which 24 are significantly upregulated in BvM and 5 are downregulated. This indicates selective deployment of individual paralogs rather than uniform activation of the transporter family. A similar pattern is observed in serine metabolism, where *serA2* (3-phosphoglycerate dehydrogenase, the first committed step of serine biosynthesis) is upregulated (BvM log_2_FC +2.57). In parallel, the chemotaxis pathway response regulator *cheY* is downregulated (BvM −1.84), consistent with a transition from motile to sessile physiology previously reported in other Proteobacteria (25). The full *liv*, *ilv*, *ser*, and *gly* paralog families are shown in Fig. S6.

To test directly whether the AA program is biofilm specific or PET driven, we plotted the BvM log_2_FC against the PvM log_2_FC for each AA-category gene (Fig. 4c). The 76 AA-category genes with finite log_2_FC in both contrasts cluster tightly along the *y* = *x* diagonal (Spearman *ρ* = +0.95), showing that AA-pathway regulation moves in the same direction whether cells are biofilm attached or planktonic on PET. The AA response is therefore a carbon-source effect rather than a biofilm-phenotype effect.

### Amino acid supplementation drives biofilm-specific biomolecular remodeling

**Fig 5:**
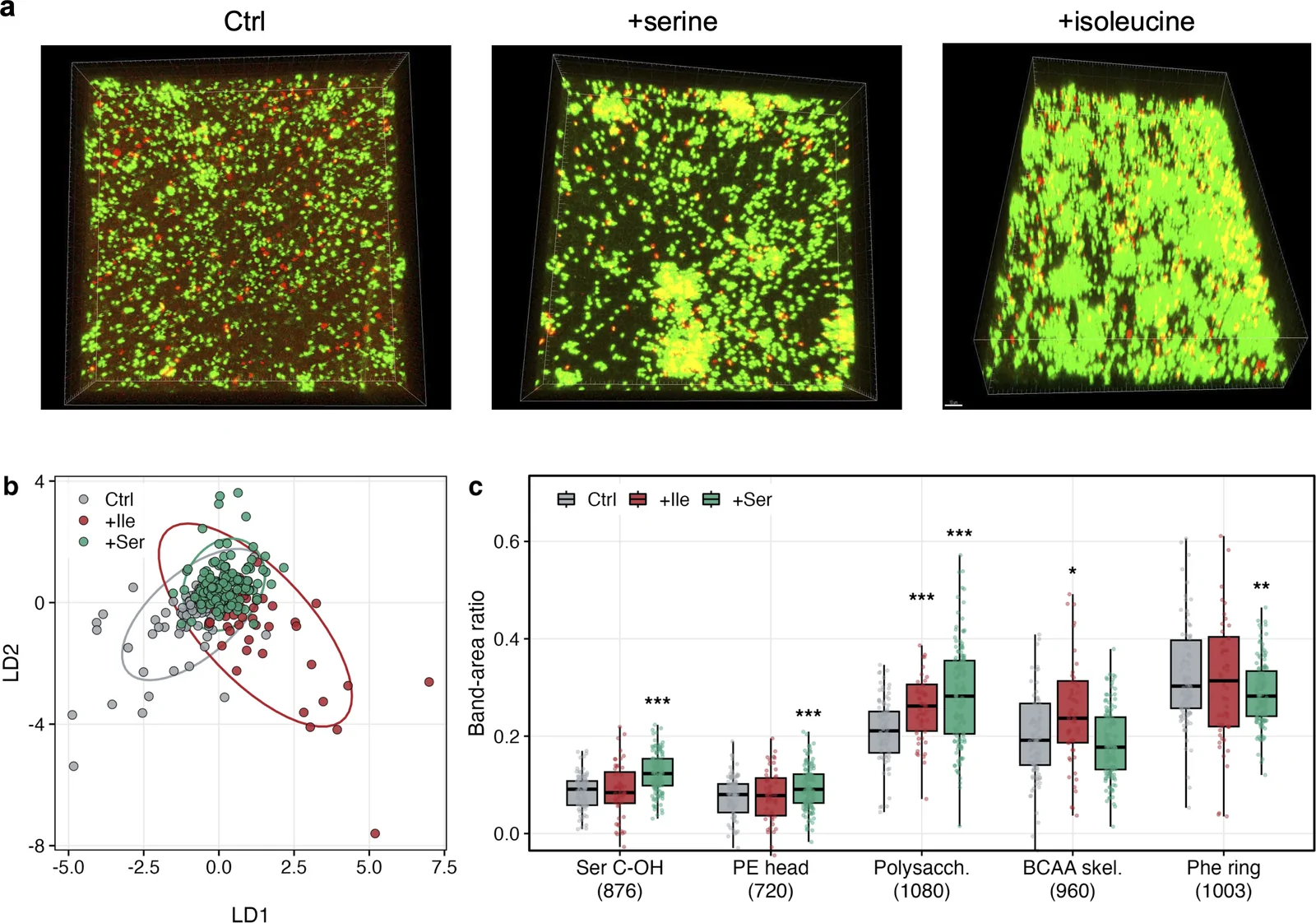
Amino acid supplementation drives biofilm-specific biomolecular remodeling that matches the transcriptomic response. (**a**) Confocal live/dead imaging of *P. sakaiensis* biofilms grown on PET in SV medium without supplementation (SV control) or supplemented with isoleucine (+Ile) or serine (+Ser); representative fields stained with SYTO9 (viable cells, green) and propidium iodide (non-viable cells, red). Imaging was performed on *n* = 2 independent biofilm cultures per condition; per-condition quantitative readouts are given in Table S3. The scale bars represent 10 μm. (**b**) Linear discriminant analysis of all 309 biofilm single-cell Raman spectra, grouped by amino acid supplementation level (Ctrl, +Ile, and +Ser). Points are individual cells’ spectra; shaded regions are 90% confidence ellipses. (**c**) Five band-area ratios computed per single-cell spectrum and pooled to box plots per AA group. Categories (left to right) mirror the Fig. 4 transcriptomic prediction: Ser C-OH (876 cm^−1^, serine incorporation fingerprint); PE (phosphatidylethanolamine) head group (720 cm^−1^); polysaccharide (1,080 cm^−1^); BCAA skeletal (960 cm^−1^, branched-chain aliphatic C–C vibration); and Phe ring breath (1,003 cm^−1^, bulk protein content proxy). Box plots show median and interquartile range; whiskers extend to 1.5 × IQR. Asterisks mark within-biofilm two-sided Wilcoxon rank-sum test *q* values (Benjamini–Hochberg corrected) for each Ctrl-vs-supplementation comparison (*q < 0.05, **q < 0.01, ***q < 0.001).

To test whether the AA pathways flagged are functionally load bearing, we perturbed them exogenously by supplementing biofilm cultures with individual amino acids. *P. sakaiensis* biofilms were grown on PET in SV medium alone (control) or supplemented with valine (Val), isoleucine (Ile), or Ser. Confocal live/dead imaging revealed supplementation-specific phenotypes (Fig. 5a, full 3D view in Fig. S7, per-condition summary in Table S3). Relative to SV control biofilms (2.8% SYTO9^+^ area coverage; 97.5% viable surfaces), +Val biofilms collapsed to a viable fraction of 21.9%, a 75.6 percentage-point drop, with the membrane-compromised cells clustering as SYTO9^+^ PI^+^ rather than PI^+^ alone, consistent with disruption of the tightly regulated BCAA-import balance highlighted above. +Ile biofilms exhibited SYTO9^+^ coverage 5.2-fold that of the control (14.7% vs 2.8% of field area) while maintaining 99.9% viability. +Ser biofilms reached 4.4-fold coverage (12.5%) at 99.7% viability, consistent with the serA2 upregulation in BvM and the polysaccharide-matrix densification reported below.

To resolve supplementation effects at single-cell resolution, we acquired Raman spectra from biofilm-derived cells under the three conditions with sufficient viable biofilms (control, +Ile, +Ser; *n* = 309 biofilm spectra in total). Valine was omitted because the +Val biofilm was extensively membrane compromised (only 21.9% viable surfaces, Table S3), leaving few biofilm-attached cells to sample at single-cell Raman throughput (Fig. 5a). PCA followed by linear discriminant analysis on the first 20 principal components separated the three AA groups with 69.3% accuracy under stratified fivefold cross-validation against a chance rate of 33.3% (Fig. 5b), demonstrating that AA supplementation induces reproducible, single-cell-resolvable biochemical signatures within biofilms.

To localize this signature to specific biomolecular pools, we computed five band-area ratios per spectrum, each normalized to the cell-size-insensitive CH-deformation band at 1,450 cm^−1^ (Fig. 5c). The five numerator bands target different molecular pools (26): serine incorporation (Ser C–OH stretch at 876 cm^−1^), the phosphatidylethanolamine head group (720 cm^−1^), polysaccharide-matrix density (C–O–C glycosidic stretch at 1,080 cm^−1^), branched-chain amino acid accumulation (BCAA skeletal C–C stretch at 960 cm^−1^), and bulk protein content (phenylalanine ring-breathing mode at 1,003 cm^−1^). Serine supplementation raised Ser C–OH (Cliff’s *δ* = +0.51, *q* = 5 × 10^−12^), the PE head-group band (*δ* = +0.25, *q* = 5 × 10^−4^), and the polysaccharide band (*δ* = +0.40, *q* = 6 × 10^−8^) and lowered the Phe ring band (*δ* = −0.20, *q* = 5 × 10^−3^). Isoleucine supplementation raised the BCAA skeletal band (*δ* = +0.21, *q* = 3 × 10^−2^) and the polysaccharide band (*δ* = +0.37, *q* = 1 × 10^−4^). Each supplemented amino acid raises its predicted biomolecular pool, consistent with (rather than demonstrating) the bulk transcriptomic predictions of Fig. 4 at single-cell resolution. These supplementation experiments therefore provide physiological support for the transcriptomic predictions rather than direct mechanistic proof: because added amino acids also perturb global metabolism, they do not on their own establish a direct causal link between individual differentially expressed genes and the biofilm phenotype.

### Biofilm cells specifically upregulate tripartite tricarboxylate transporters from three distinct genomic loci

**Fig 6:**
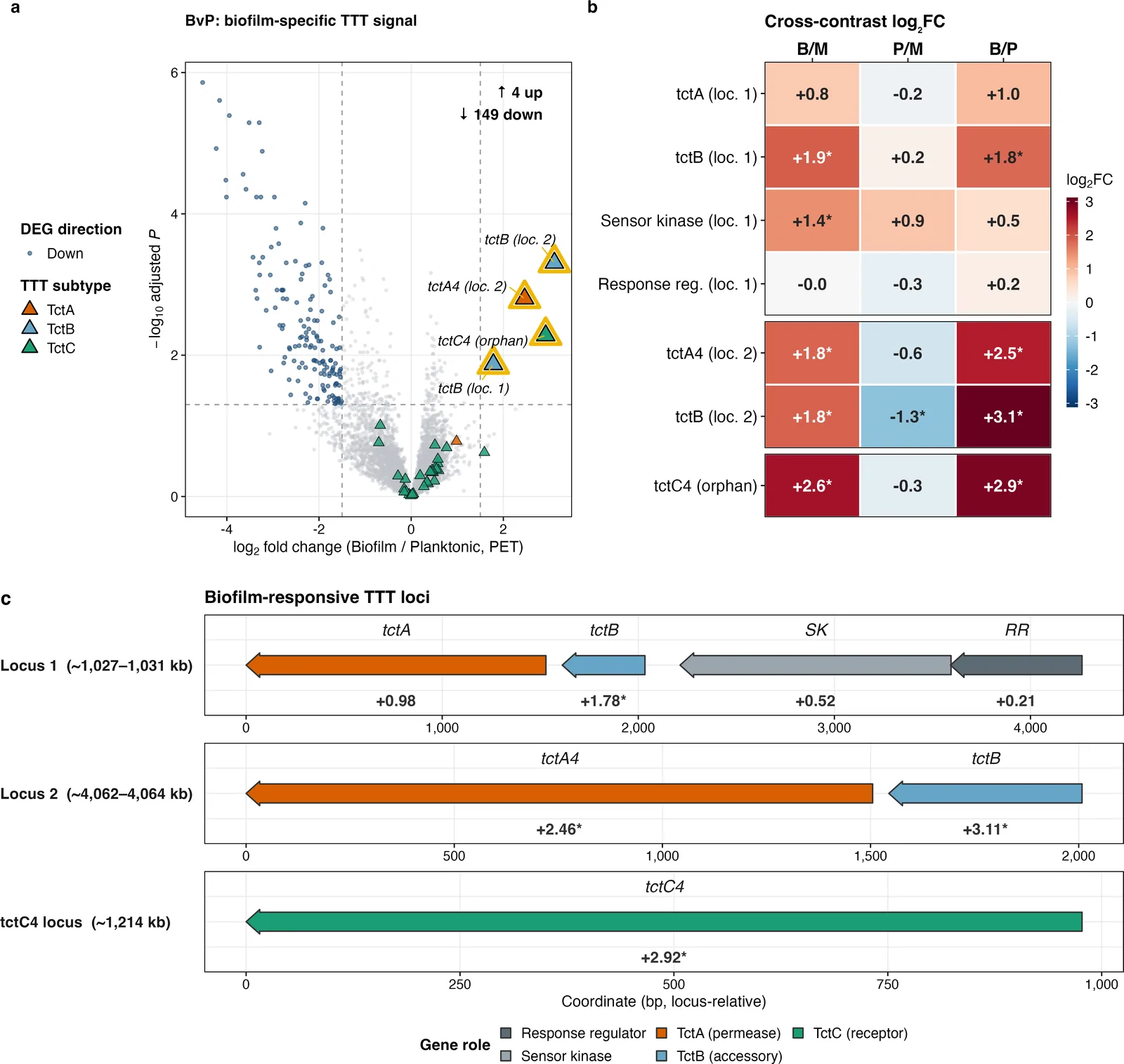
Biofilm cells specifically upregulate tripartite tricarboxylate transporters from three distinct genomic loci. (**a**) Volcano plot of the biofilm-vs-planktonic on PET (BvP) contrast (threshold *P* adj <0.05 and |log_2_FC| >1.5: 4 up/149 down). All 33 TTT-family genes encoded by the *P. sakaiensis* genome are overlaid as triangles colored by subtype (TctA, TctB, and TctC); the four biofilm-induced TTT genes are outlined in gold. (**b**) Cross-contrast log_2_FC heatmap for seven TTT-context genes: the four biofilm-induced hits *tctB* (loc. 1), *tctA4* and *tctB* (loc. 2), and *tctC4*, plus the locus 1 operon mates *tctA* (loc. 1), sensor kinase, and response regulator. Rows grouped by genomic locus (locus 1 at 1,027–1,031 kb; locus 2 at 4,062–4,064 kb; orphan *tctC4* at 1,214 kb). Cell values are signed log_2_FC; asterisks denote *P* adj <0.05. (**c**) Genomic organization of the three biofilm-responsive TTT loci. Arrow fill indicates gene role, and direction reflects the annotated strand (all seven CDSs on the minus strand). Italic gene symbols above each arrow; signed BvP log_2_FC with *P* adj <0.05 asterisks below. Locus-relative base pair coordinates on the *x*-axis (each track independently normalized to start at 0).

We next examined the biofilm-specific transcriptional program on PET using the BvP contrast. This comparison revealed a highly asymmetric distribution of differentially expressed genes: only 4 of the 153 BvP DEGs were upregulated in biofilm cells, whereas 149 were downregulated (Fig. 6a). Strikingly, all four upregulated genes belong to the TTT family.

*P. sakaiensis* encodes 33 TTT-family genes genome-wide (KEGG orthology assignments K07795–K07797 on the NCBI annotation of assembly CP166677 [see the supplemental methods]), but only 4 of these (12%) are induced in biofilm cells, while the remaining 29 show no significant change in the BvP contrast. All 33 TTT genes are overlaid as triangles on the BvP volcano (Fig. 6a), with the four induced ones highlighted. This selective response argues against a global increase in TTT activity and instead indicates targeted activation of specific transporter modules. The full 33-gene × 3-contrast heatmap is shown in Fig. S8.

All four biofilm-induced TTT genes are elevated in BvM and BvP but flat or negative in PvM (Fig. 6b), the signature of a biofilm-specific upregulation that collapses when the biofilm phenotype is removed on the same PET substrate. The four genes localize to three distinct genomic regions with different operon architectures (Fig. 6c). Locus 1 (~1,027 to 1,031 kb, minus strand) is a complete TTT operon paired with a two-component signaling module across four contiguous coding sequences: the TTT permease *tctA*, the TTT accessory *tctB*, a sensor histidine kinase, and a response regulator. Only *tctB* reaches the biofilm-specificity threshold in BvP (log_2_FC +1.78, *P* adj = 1.3 × 10^−2^), while *tctA* rises together but falls below the |log_2_FC| >1.5 cutoff (+0.98, ns). The response regulator is essentially constitutive across all three contrasts (|log_2_FC| ≤0.3, all ns); the sensor kinase is PET-responsive (BvM log_2_FC +1.44, *P* adj = 2.1 × 10^−3^) but not biofilm specific (BvP +0.52, ns). The operon therefore behaves as a pre-configured module: a resident response regulator and a PET-sensing kinase are in place across conditions, while transporter output is additionally gated by the biofilm phenotype. Locus 2 (~4,062 to 4,064 kb, minus strand) is a minimal two-gene cassette: *tctA4* (BvP log_2_FC +2.46, *P* adj = 1.6 × 10^−3^) and a second *tctB* paralog (BvP +3.11, *P* adj = 4.9 × 10^−4^), with only 38 bp between coding sequences and no cis-encoded regulator. The orphan *tctC4* (~1,214 kb, minus strand) is a stand-alone Bug-family substrate-binding protein (BvP log_2_FC +2.92, *P* adj = 5.3 × 10^−3^), located ~0.19 Mb downstream of locus 1 and ~2.85 Mb upstream of locus 2 on the same chromosome but contiguous with neither operon.

Placing these genes in the context of the NCBI coordinate annotation of assembly CP166677 links them to the terephthalate branch of the pathway. The Bug-family substrate-binding protein *tctC* (ABFT21_17730) lies inside the *tph* terephthalate 1,2-dioxygenase cluster (ABFT21_17715–17750) and is among the most strongly PET-induced genes in the genome (BvM log_2_FC +5.0, *P* adj = 7.4 × 10^−12^; PvM +3.4, *P* adj = 3.8 × 10^−7^), rising in parallel with *tphA1–A3*, *tphB*, PETase, and MHETase but flat in BvP (+1.6, ns; Table S1). Because substrate specificity in the TTT architecture is set by the periplasmic binding-protein component (27, 28), the substrate-recognition element sitting with the terephthalate ring-cleavage operon is induced as part of the shared PET response, whereas the membrane components induced specifically in biofilm cells, *tctA4* and *tctB* at locus 2 (ABFT21_18265 and ABFT21_18270) and *tctB* at locus 1 (ABFT21_04760), together with the orphan Bug-family binding protein *tctC4* (ABFT21_05640), add a phenotype-gated transport step on top of it. Biofilm cells therefore selectively upregulate transport components of a pathway whose catabolic and substrate-binding machinery is a general PET response.

Tripartite tricarboxylate transporters have been implicated in TPA uptake in the related β-proteobacterium *Comamonas* sp. E6 (29). Given that *P. sakaiensis* degrades PET into TPA and EG via PETase and MHETase, the biofilm-specific TTT mobilization reported here is therefore consistent with a putative role in substrate capture at the polymer interface, potentially facilitating uptake of PET-derived intermediates such as TPA. No transporter-activity, uptake, or mutant assay was performed in this study, so this assignment rests on transcriptional and comparative-genomic evidence and remains correlative; alternative TTT substrates are discussed below.

### Biofilm cells exhibit a single-cell membrane-composition phenotype

The four biofilm-induced TTT components identified in the previous section are dominated by integral-membrane subunits: the TctA permease in locus 2 and both TctB accessory proteins are embedded in the inner membrane, while the orphan TctC4 is the periplasmic substrate-binding partner. Preferential mobilization of membrane-spanning transporters is often accompanied by compensatory remodeling of the surrounding bilayer to accommodate the increased transmembrane-protein load, typically reflected in a shift toward more ordered and saturated lipid acyl chains. To assess whether such a phenotype is present, we therefore examined single-cell Raman fingerprints of biofilm and planktonic populations.

**Fig 7:**
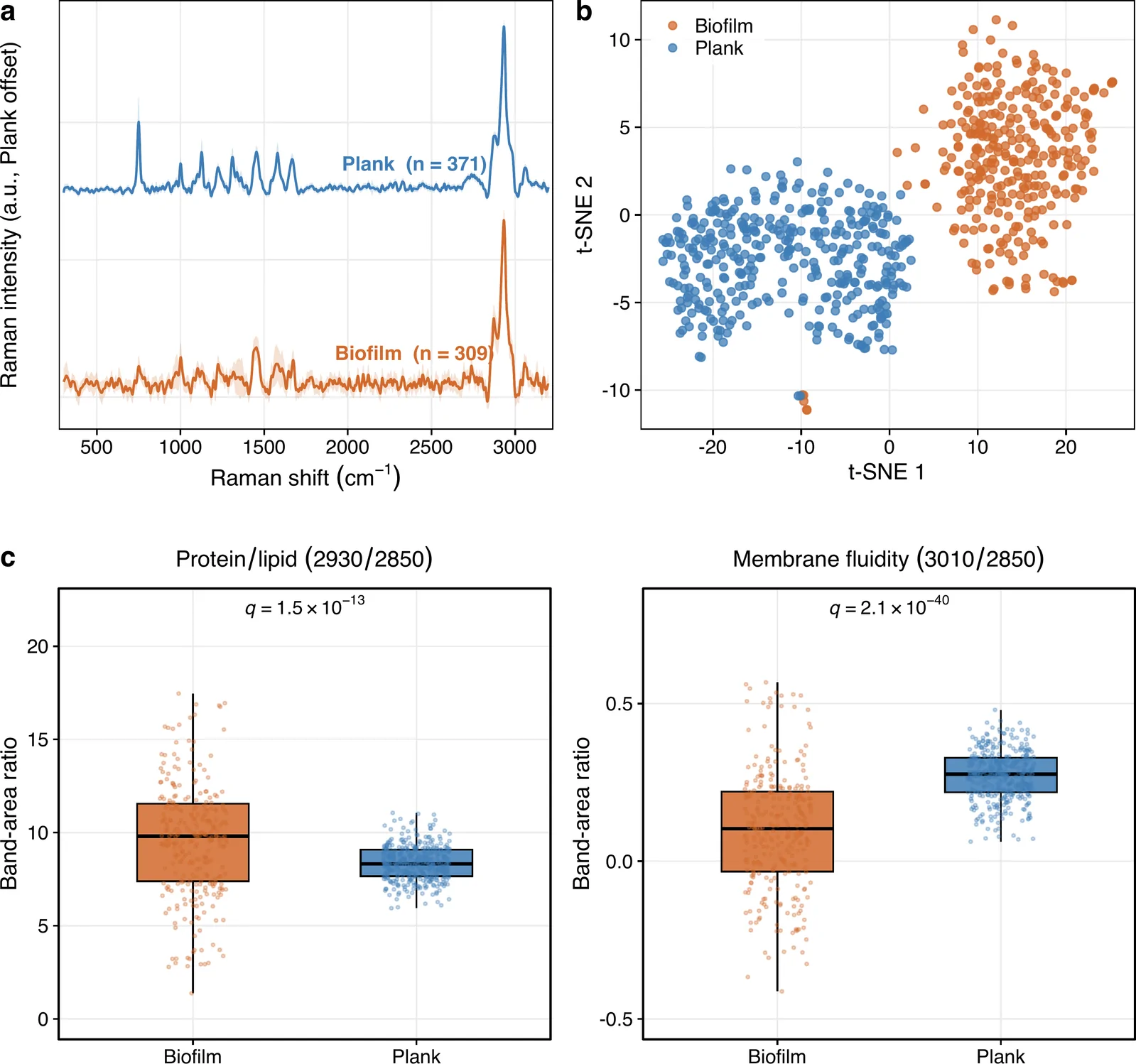
Biofilm cells exhibit a single-cell membrane-composition phenotype consistent with TTT upregulation. (**a**) Mean Raman spectra of *P. sakaiensis* cells harvested as biofilm from the PET surface (*n* = 309 single-cell spectra) or as planktonic cells from the same culture (*n* = 371), each pooled across the three amino acid supplementation conditions (control, +Ile, and +Ser). (**b**) t-Distributed stochastic neighbor embedding (t-SNE) of the same 680 spectra, colored by phenotype. (**c**) Trapezoidally integrated CH-stretch band-area ratios pooled into the binary biofilm (*n* = 309) vs planktonic (*n* = 371) contrast. (Top) Protein-to-lipid ratio (CH_3_ asymmetric stretch at 2,930 cm^−1^ divided by CH_2_ symmetric stretch at 2,850 cm^−1^). (Bottom) Membrane fluidity ratio (=CH *cis* stretch at 3,010 cm^−1^ divided by CH_2_ symmetric stretch at 2,850 cm^−1^). Two-sided Wilcoxon rank-sum test, Benjamini–Hochberg corrected; *q < 0.05, **q < 0.01, ***q < 0.001. The equivalent six-group breakdown, showing that the phenotype difference is preserved within each amino acid supplementation condition, is given in Fig. S9.

Pooled across the three AA-supplementation conditions, the mean spectra of biofilm-derived cells (*n* = 309) and planktonic cells (*n* = 371) on PET differ visibly across multiple biomarker regions (Fig. 7a). Planktonic cells are elevated at the cytochrome c ν_15_ band (750 cm^−1^) and the amide I region (~1,660 cm^−1^), consistent with higher respiratory and protein-synthesis demand in the metabolically active planktonic phenotype, while biofilm cells are elevated in the CH-stretch region (~2,872 cm^−1^), suggesting altered lipid–protein balance in the membrane fraction.

t-SNE embedding of all 680 spectra resolves biofilm and planktonic cells into two distinct clusters (Fig. 7b), indicating a robust phenotype-level distinction. To resolve the CH-stretch signal into interpretable membrane descriptors, we computed two band-area ratios per spectrum (Fig. 7c): a protein/lipid ratio (CH_3_ asymmetric stretch at 2,930 cm^−1^ relative to CH_2_ symmetric stretch at 2,850 cm^−1^) and a membrane fluidity proxy (=C–H *cis* stretch at 3,010 cm^−1^ relative to the same CH_2_ reference, reflecting unsaturated acyl content). Pooled across the three AA conditions, biofilm cells carry a significantly higher protein/lipid ratio (*q* = 1.5 × 10^−13^, Cliff’s *δ* = +0.33; Fig. 7c) and markedly lower unsaturated acyl content (*q* = 2.1 × 10^−40^, Cliff’s *δ* = −0.59) than planktonic cells, indicating a more protein-rich and more saturated membrane environment. The six-group breakdown in Fig. S9 confirms that this distinction is preserved across each AA condition. The physical signature is consistent with integral-membrane transporters being preferentially mobilized and acyl chains saturating to accommodate them, providing an independent single-cell correlate of the biofilm-specific TTT upregulation in Fig. 6.

Together, these observations define a two-layer model of the *P. sakaiensis* PET response (Fig. S10): a carbon-source-driven program shared by all PET-exposed cells (layer 1) and a biofilm-specific mobilization of TTT transporters (layer 2), with the single-cell membrane phenotype as an independent readout.

## DISCUSSION

Three-way DESeq2 analysis across biofilm-PET, planktonic-PET, and planktonic-maltose conditions resolves the *P. sakaiensis* PET response into two metabolically distinct layers, only one of which is biofilm specific. This design separates carbon-source effects from phenotype effects, avoiding the confounding inherent in standard two-condition comparisons.

The first layer is a carbon-source-driven response shared between biofilm and planktonic cells on PET: it spans the entire PET-catabolic route, with the PETase and MHETase hydrolases, the terephthalate ring-cleavage operon, and the protocatechuate branch coordinately induced on PET in both phenotypes (Fig. 3b and c), together with a genome-wide lifestyle shift in which flagellar, chemotaxis, and TCA-cycle genes fall while exopolysaccharide-matrix and membrane-lipid genes rise (Fig. S5), and is dominated in the upregulated direction by amino acid transport and metabolism (BvM-vs-PvM Spearman *ρ* = +0.95 across 76 AA-category genes; Fig. 4c). Within this shared layer, the BCAA transcriptional pattern is paradoxical at the level of carbon flux. Twenty-four of 29 *livKHMGF*-type ABC-transporter paralogs are upregulated in PET cells, while *ilvE*, the first committed step of BCAA catabolism, is suppressed in both BvM and PvM (Fig. 4). If imported BCAAs were destined for bulk catabolism, a modest elevation of valine should be neutral or beneficial; instead, +Val biofilms are dominated by membrane-compromised cells (only 21.9% viable surfaces; Table S3; Fig. 5a). The phenotypic constraint argues against a bulk-uptake-for-energy model and points to a tightly constrained import–utilization balance. Paralog selectivity reinforces the same conclusion: the *livKHMGF* family does not move uniformly, with 24 paralogs up and 5 down in the same contrast, the signature of fine-grained operon-level regulation rather than blanket family-wide activation. Individual BCAAs act as biofilm-promoting environmental cues in other Proteobacteria, with isoleucine, leucine, and valine each elevating intracellular c-di-GMP and repressing swarming motility in *Pseudomonas aeruginosa* (30), so the *P. sakaiensis* import response is most parsimoniously read as cells acquiring intracellular BCAA pools without committing them to catabolic flux. Single-cell Raman localizes this material to the matrix-building fraction: +Ile biofilms attain the highest SYTO9^+^ area coverage of any supplementation condition (5.2-fold the SV control; Table S3; Fig. 5a), with a coordinated rise in both the BCAA skeletal band and the polysaccharide-matrix band per cell (Fig. 5c), consistent with imported BCAAs incorporated into matrix-resident proteins.

Serine metabolism in PET-grown cells, also part of layer 1, shows a transcriptional signature of motile-to-sessile transition: *serA2*, the first committed step of serine biosynthesis, is strongly upregulated, while *cheY*, the master chemotaxis response regulator, is downregulated (Fig. 4). The combination of *de novo* serine production with general chemotaxis suppression fits the broader switch toward biosynthetic self-sufficiency and surface attachment that defines the sessile, matrix-producing state in environmental Proteobacteria (15). Serine supplementation confirms that availability is rate-limiting for this transition: serine-supplemented biofilms reach 4.4-fold the SYTO9^+^ area coverage of the control at undiminished viability (12.5% vs 2.8%; 99.7% viable surfaces; Table S3; Fig. 5a). Single-cell Raman of biofilm cells under +Ser reports four coordinated effects (Fig. 5c). The Ser C–OH band at 876 cm^−1^ rises strongly (Cliff’s *δ* = +0.51, *q* = 5 × 10^−12^), confirming direct serine incorporation. The polysaccharide-matrix band at 1,080 cm^−1^ rises in parallel (*δ* = +0.40, *q* = 6 × 10^−8^), the single-cell signature expected when added serine fuels biosynthesis of matrix exopolysaccharide and provides the per-cell mechanism linking *serA2* upregulation to the denser, matrix-rich +Ser biofilm phenotype. The phosphatidylethanolamine head-group band rises more modestly (*δ* = +0.25, *q* = 5 × 10^−4^), an empirical observation consistent with serine flux into PE biosynthesis via phosphatidylserine and bacterial phosphatidylserine decarboxylase (31), though direct evidence for the rerouting requires lipidomics that is beyond the scope of this work. The phenylalanine ring-breathing band drops modestly (*δ* = −0.20, *q* = 5 × 10^−3^), consistent with bulk protein content falling relative to other biomass pools as carbon is redirected into serine-derived destinations.

Superimposed on this shared metabolic reprogramming is a second, sharply selective layer that is specific to the biofilm phenotype. When carbon source is held constant (BvP contrast), only 4 of 33 TTT-family genes are upregulated, while the majority show no change (Fig. 6 and Fig. S8). This selectivity argues for substrate-matched mobilization of specific carriers from a much larger paralog pool rather than a non-specific increase in transport capacity. The TTT family is a binding-protein-dependent secondary-transporter superfamily broadly distributed in bacteria, with substrate specificity set at the periplasmic-binding-protein component and a scope spanning citrate, dicarboxylates, and aromatic acids (27, 32). This finding extends the conventional view that biofilms accelerate plastic degradation primarily by retaining hydrolytic enzymes near the polymer surface within the extracellular matrix (15). In *P. sakaiensis*, biofilm formation is associated with the selective mobilization of a small, specific set of transporters, consistent with the enhanced capture of aromatic degradation products released by these enzymes.

Alternative substrates should nonetheless be considered. The TTT family is functionally broad: characterized members transport citrate, C_4_-dicarboxylates such as malate and succinate, and a range of aromatic acids in addition to terephthalate (27, 28, 32). The Bug-family binding proteins that set specificity are among the most expanded paralog families in β-proteobacteria, and *P. sakaiensis* encodes 33 TTT genes, so an assignment based on expression alone cannot exclude that the biofilm-induced carriers handle citrate, a C_4_-dicarboxylate, or a different PET-derived aromatic such as protocatechuate. Terephthalate remains the best-supported candidate: it is the quantitatively dominant aromatic released at the polymer surface; the *tph* ring-cleavage operon and its cognate *tctC* binding protein are strongly co-induced on PET (Table S1); and the closest functional precedent is TPA uptake by the TTT system of *Comamonas* sp. E6 (29). It is offered here as the leading hypothesis rather than an established function; discriminating among these possibilities will require uptake assays with labeled substrates in transporter-deletion backgrounds, which were not part of this study.

The four induced genes localize to three distinct genomic regions with different operon configurations. Locus 1 is a complete TctA/TctB transporter paired with a two-component signaling module in which the response regulator is constitutive and the sensor kinase is PET responsive but not biofilm specific, while only the transporter components are biofilm gated; this “primed operon” pattern, with PET sensing always on across PET-exposed conditions and transport additionally gated by phenotype, places biofilm-specific upregulation downstream of PET sensing. A direct precedent exists in the *Bordetella pertussis* TctCBA system, where the TTT periplasmic binding protein BctC interacts with the cognate sensor kinase BctE of an adjacent two-component system to upregulate the transporter operon in the presence of substrate (28). Locus 2 is a minimal transporter cassette in which both genes (*tctA4* and a second *tctB* paralog) clear the biofilm-specificity threshold with no cis-encoded regulator, and the orphan *tctC4* sits ~0.19 Mb from locus 1 with no contiguous transporter, so any functional pairing must be in *trans*. The three loci therefore look more like parallel recruitment of pre-existing scaffolds than a single co-regulated regulon.

Several limitations should be considered. The three-way design lacks a matched non-PET biofilm control, so some signatures attributed to the biofilm phenotype could in part reflect surface attachment under this particular PET regime rather than PET exposure *per se*. Because current data derive from mature 32-day biofilms, future time-resolved analysis is needed to resolve whether amino acid reprogramming and transporter induction emerge progressively during development or represent a strictly mature state. In addition, the proposed role of the induced TTTs in TPA uptake remains correlative. Direct validation will require targeted genetic perturbations, including genetic knockouts and uptake assays using labeled substrates. Additional experiments, including EPS proteomics, would further test whether imported BCAAs and serine are incorporated into matrix-associated proteins. Finally, although coordinate reannotation against the NCBI RefSeq annotation of assembly CP166677 resolves the PET-catabolic and transporter genes to defined functions, a substantial set of PET-responsive genes is still annotated only as hypothetical or domain-of-unknown-function proteins; these uncharacterized, strongly PET-regulated genes are the most obvious candidates for future functional study.

In conclusion, this work resolves the *P. sakaiensis* response to PET into a two-layer metabolic model: a shared amino acid reprogramming across all PET-exposed cells, and a highly selective, biofilm-specific mobilization of TTT transporters from distinct genomic loci, accompanied by a corresponding single-cell membrane phenotype. The combined transcriptomic and single-cell Raman evidence is consistent with a putative role for these biofilm-specific transporters in capturing the TPA released by PETase and MHETase at the polymer surface. This framework extends the current understanding of biofilm function in plastic degradation and provides a testable basis for linking transporter activity to substrate capture at the polymer interface.

## MATERIALS AND METHODS

### Strain and culture conditions

*Piscinibacter sakaiensis* 201-F6 (NBRC 110686) was obtained lyophilized from the Bioresource Collection and Research Center. It was reconstituted using NBRC 802 medium (10 g/L hipolypeptone [Wako Pure Chemical Corporation], 2 g/L yeast extract, and 1 g/L MgSO_4_·7H_2_O) at 30°C. The culture was then streaked onto NBRC 802 agar for purity before storing in glycerol. *P. sakaiensis* glycerol stock was then streaked on NBRC 802 with the addition of agar (pH 7). Single colonies appeared after 4 days. A single colony was then inoculated into NBRC 802 liquid medium and cultured for 2 days at 30°C at 200 rpm.

Experimental cultures were grown in chemically defined SV medium (16), formulated without yeast extract to keep the carbon inventory chemically defined [composition per liter: NaHCO_3_ 202 mg, (NH_4_)_2_SO_4_ 1,011 mg, CaCO_3_ 101 mg, thiamine·HCl 2.53 mg, biotin 50.6 µg, vitamin B_12_ 0.506 mg, FeSO_4_·7H_2_O 10.1 mg, MgSO_4_·7H_2_O 10.1 mg, CuSO_4_·5H_2_O 1.01 mg, MnSO_4_·H_2_O 0.709 mg, ZnSO_4_·7H_2_O 1.01 mg in 10 mM phosphate buffer adjusted to pH 7]. Vitamins and trace elements were added from concentrated stocks: the vitamin stock contained 125 mg thiamine·HCl, 2.5 mg biotin, and 25 mg vitamin B_12_ in 50 mL water and was added at 1.011 mL/L, and the trace-element stock contained 100 mg FeSO_4_·7H_2_O, 100 mg MgSO_4_·7H_2_O, 10 mg CuSO_4_·5H_2_O, 7.01 mg MnSO_4_·H_2_O, and 10 mg ZnSO_4_·7H_2_O in 100 mL water and was added at 10.11 mL/L. PET films were supplied as the sole carbon source, as described in the next section. *P. sakaiensis* was inoculated at an initial OD_600_ of 0.2 into 50 mL cultures containing pre-sterilized 22 × 22 mm PET coupons and incubated at 30°C without shaking for 32 days prior to harvest of biofilm and planktonic fractions. The static regime was chosen to prevent mechanical detachment of the nascent biofilm from the PET surface.

For amino acid supplementation experiments, SV medium was supplemented with L-valine (0.05 mg/mL), L-isoleucine (0.05 mg/mL), or L-serine (0.16 mg/mL) (33, 34) sterilized with 0.22 µm pore-size filters.

Biofilm cells were harvested directly from PET surfaces, while planktonic cells were collected from the corresponding culture supernatants. Prior to downstream processing, PET coupons were gently washed with sterile PBS to remove loosely attached cells.

For RNA sequencing, biofilm-associated cells on PET films were processed directly by placing the entire PET coupon into lysing matrix tubes from the SPINeasy RNA Kit for Bacteria (MP Biomedicals), followed by mechanical disruption according to the manufacturer’s protocol. Planktonic cells were harvested from the culture supernatant by centrifugation prior to RNA extraction.

### PET substrate and degradation assays

Amorphous PET film (1.9% crystallinity, thickness 0.25 mm; GoodFellow Advanced Materials) was cut into 22 × 22 mm coupons, sterilized by immersion in 70% ethanol overnight, and dried in a biosafety cabinet for 1 h prior to inoculation. Coupons were pre-weighed on an analytical balance (precision ± 0.0001 g). Sterilized PET coupons were transferred aseptically into SV medium culture flasks as described in the previous section. Detailed methods for characterization of PET and RNA extraction are provided in the supplemental material.

### Single-cell Raman spectroscopy

Two single-cell Raman experiments are reported in this study. In experiment 1, *P. sakaiensis* was harvested from planktonic cultures on maltose (control carbon source, *n* = 73 single-cell spectra) or on PET as the sole carbon source (*n* = 58), a two-group comparison of carbon-source-induced metabolic state. In experiment 2, biofilm and planktonic cells from PET cultures with or without amino acid supplementation were analyzed in a six-group factorial design ([biofilm, planktonic] × [Ctrl, +Ile, +Ser]; total *n* = 680 spectra, comprising Biofilm_Ctrl *n* = 110, Biofilm_Ile *n* = 56, Biofilm_Ser *n* = 143, Plank_Ctrl *n* = 85, Plank_Ile *n* = 152, and Plank_Ser *n* = 134). Cells were harvested from the biofilm fraction (PET surface) or planktonic fraction (supernatant) and washed three times in distilled water to remove residual culture medium and extracellular metabolites. Cell suspensions were diluted in distilled water until individual cells could be resolved at the microscope; a 1.5 µL aliquot of the diluted suspension was then deposited on an aluminum-coated slide and air-dried at room temperature prior to spectroscopic measurement.

Single-cell Raman spectra were recorded on a custom-built Raman spectrometer fitted with a 50× air objective and a 300 grooves/mm diffraction grating spanning 232–3,621 cm^−1^. Excitation was provided by a 532 nm laser attenuated through neutral-density filters to ~6 mW at the sample plane. Integration time was 3 s per spectrum per cell. Retained spectra cover 301–3,199 cm^−1^ across 1,335 channels (step size ≈ 2.13 cm^−1^) after edge-channel trimming at the QC step. Further details of the Raman analysis are provided in the supplemental material.

### Confocal live/dead microscopy

Biofilm samples grown on PET in SV medium, with or without amino acid supplementation, were gently rinsed with sterile PBS prior to staining. Samples were stained using the LIVE/DEAD BacLight Bacterial Viability Kit (Thermo Fisher Scientific), with SYTO9 (5 µM) and propidium iodide (PI, 30 µM) prepared in 0.9% NaCl. Staining was performed in the dark for 30 min.

Confocal imaging was performed on *n* = 2 independent biofilm cultures per condition. Z-stack images were acquired using a Zeiss LSM 780 confocal laser-scanning microscope equipped with a 63× oil immersion objective. SYTO9 and PI were excited at 488 and 561 nm, respectively. Image reconstruction and analysis were performed using Imaris software (Bitplane, Oxford Instruments). Per-condition quantitative readouts (Table S3) were derived as follows. For SYTO9^+^ area coverage, the maximum-intensity projection of each z-stack was cropped to the imaged field of view, and the green (SYTO9) channel was thresholded by Otsu’s method with an 8-bit floor of 60 digital number units to exclude autofluorescence and chromatic ringing; coverage is reported as the fraction of in-field pixels above threshold. For the viable surface fraction, the Imaris intensity mean statistic was exported per surface object for both channels, and each surface was classified using fixed intensity thresholds anchored to the SV-control intensity distribution (SYTO9 >30, PI >50): viable (SYTO9^+^ PI^–^), membrane-compromised (SYTO9^+^ PI^+^), or non-viable (SYTO9^–^ PI^+^). Viable surface fraction is the count of viable surfaces divided by the count of classified (i.e., non-background) surfaces.

### Statistical analysis

Unless otherwise stated, two-group comparisons used two-sided Wilcoxon rank-sum tests, and multi-group comparisons used Kruskal–Wallis or one-way ANOVA, where group variances were homogeneous; effect sizes are reported as Cliff’s *δ*. Both per-wavenumber spectral tests (within each contrast) and the summary biomarker band-area-ratio tests (within each figure’s test family) were Benjamini–Hochberg-corrected, and cross-contrast log_2_FC correlations were summarized by Spearman *ρ*. A random seed of 42 was used throughout to guarantee reproducible dimensionality reduction and cross-validation fold assignment.

## Data Availability

RNA-seq data for the 15 libraries (biofilm-PET, planktonic-PET, planktonic-maltose; *n* = 5 per condition) have been deposited at Figshare (https://doi.org/10.6084/m9.figshare.32548194). The single-cell Raman spectra (131 spectra from the PET-exposure pair and 680 spectra from the phenotype × amino acid supplementation cohort) are available at Figshare (https://doi.org/10.6084/m9.figshare.32548938). All deposited data sets are released under a CC BY 4.0 license.

## SUPPLEMENTAL MATERIAL

Supplemental material aem.01465-26-s0001.docx Fig. S1 to S10, Tables S1 to S3, and supplemental methods.

ASM does not own the copyrights to Supplemental Material that may be linked to, or accessed through, an article. The authors have granted ASM a non-exclusive, world-wide license to publish the Supplemental Material files. Please contact the corresponding author directly for reuse.
