# Supplementary material for: Amino acid reprogramming and biofilm-specific tricarboxylate transporters in PET-degrading Piscinibacter sakaiensis
Source: Appl Environ Microbiol. 2026 Sep 8;92(10):e01465-26. doi: 10.1128/aem.01465-26 (PMC13688920; doi:10.1128/aem.01465-26)
Supplement: Supplementary file 1 [file aem.01465-26-s0001.docx]

## **Supplementary Figures**

### **Supplementary Figure S1.** Evolved-gas Gram–Schmidt profile across the full thermal ramp.

Gram–Schmidt root-mean-square (RMS) trace of the total IR absorbance of evolved gases as a function of furnace temperature for the same recovered PET films as Fig 1c,d (PET only; PS + PET). The shaded band (380–500 °C) marks the principal polymer-decomposition region used to compute an integrated PS / PET evolved-gas ratio (annotated in-panel).

### **Supplementary Figure S2.** SEM images of the surface of (a) PET and (b) PET after incubation with *P. sakaiensis*. The scale bars represent 1 μm.

### **Supplementary Figure S3**. Sample-to-sample Spearman correlation confirms replicate coherence.

Heatmap of Spearman rank correlation coefficients across all pairwise combinations of the 15 RNA-seq libraries, computed on log_2_-normalised DESeq2 counts. Rows and columns ordered by hierarchical clustering (complete linkage on 1 − ρ). Condition annotation on the axes matches Fig 3a.

### **Supplementary Figure S4. The PET-catabolic pathway is also induced in planktonic cells on PET.** Volcano plot of the planktonic-PET-versus-maltose (PvM) contrast (DESeq2 Wald test; padj < 0.05 and |log_2_FC| > 1.5). The PET-catabolic pathway genes highlighted in Fig. 3b are overlaid and coloured by module (PET hydrolase; TPA ring catabolism; protocatechuate ortho-cleavage; EG pathway; biofilm-specific TTT transporters); PETase, MHETase and the *tctC* (TPA) substrate-binding protein are labelled. The hydrolase, TPA-ring and protocatechuate modules are up in planktonic PET cells just as in biofilm cells (Fig. 3b,c), confirming that the catabolic response is carbon-source-driven and shared, whereas the biofilm-specific TTT transporters remain flat or negative in this contrast.

### **Supplementary Figure S5. A coordinated genome-wide lifestyle programme accompanies PET catabolism and is shared between biofilm and planktonic cells.** Log_2_ fold-change heatmap of representative genes from four lifestyle modules across the three contrasts (biofilm-vs-maltose, BvM; planktonic-PET-vs-maltose, PvM; biofilm-vs-planktonic-on-PET, BvP): flagellar and chemotaxis genes and the TCA cycle (downregulated on PET), and exopolysaccharide/matrix-biosynthesis and membrane-lipid-remodelling genes including cyclopropane-fatty-acid synthase (*cfa*) and lysyl-phosphatidylglycerol synthases (upregulated on PET). Cell values are signed log_2_FC; asterisks denote significance after Benjamini–Hochberg correction (padj < 0.05, < 0.01 and < 0.001 shown as one, two and three asterisks). Each module moves in the same direction in BvM and PvM but is flat in BvP, identifying it as part of the shared, carbon-source-driven layer. Full statistics for all 139 module genes are provided as Table S2.

### **Supplementary Figure S6.** Extended heatmap of all amino-acid-related gene paralog families.

Row-z-scored expression heatmap covering every paralog of *livFGHKM* (BCAA ABC transporter), *ilvA*–*ilvH* (BCAA biosynthesis and catabolism), *serA*–*serC* / *glyA* (serine–glycine metabolism), and *psd* / *psdA* (phosphatidylserine decarboxylase). Row annotation strip indicates AA subcategory (same palette as Fig 4); column order follows Fig 4b.

### **Supplementary Figure S7.** Full confocal field-of-view for each amino-acid supplementation condition. Representative fields stained with SYTO9 (viable cells, green) and propidium iodide (non-viable cells, red). Per-condition quantitative read-outs in **Table S3**. The scale bars represent 10 μm.

### **Supplementary Figure S8.** All 33 TTT-family genes across the three contrasts.

Log_2_FC heatmap for every TTT-family gene in the *P. sakaiensis* genome (n = 33) across the three DESeq2 contrasts (BvM, PvM, BvP). Rows ordered by TTT subtype (TctC → TctB → TctA, indicated on the left side bar) and within subtype by descending BvP log_2_FC. Asterisks denote padj < 0.05 per Benjamini–Hochberg correction.

**Supplementary Figure S9. Single-cell membrane band-area ratios across the full six-group amino-acid supplementation design.** Trapezoidally integrated CH-stretch band-area ratios for biofilm and planktonic cells under each supplementation condition (Ctrl, +Ile, +Ser): (a) protein/lipid ratio (CH_3_ asymmetric stretch at 2930 cm^-1^ relative to CH_2_ symmetric stretch at 2850 cm^-1^) and (b) membrane-fluidity ratio (=C–H cis stretch at 3010 cm^-1^ relative to the same CH_2_ reference). Box plots show median and interquartile range with individual cells overlaid as points. Brackets mark within-phenotype Ctrl-versus-supplementation comparisons (two-sided Wilcoxon rank-sum test, Benjamini–Hochberg corrected; q < 0.05, < 0.01 and < 0.001 shown as one, two and three asterisks). This six-group breakdown accompanies the pooled biofilm-versus-planktonic contrast in main Fig. 7 and confirms that the phenotype-level difference is preserved within each amino-acid condition.

### **Supplementary Figure S10. A two-layer model of the PET biofilm response in *Piscinibacter sakaiensis*.** Schematic summary. PET is depolymerised by PETase and MHETase to terephthalate (TPA) and ethylene glycol (EG) at the biofilm–polymer interface (left). Layer 1 (centre) is a carbon-source-driven programme shared by biofilm and planktonic cells on PET: broad amino-acid reprogramming (branched-chain-amino-acid transport up, serine biosynthesis up, *ilvE* down), suppression of chemotaxis and flagellar motility and of the TCA cycle, and induction of exopolysaccharide/matrix and membrane-lipid-remodelling genes. Layer 2 (right) is biofilm-specific: selective induction of TTT transporters, comprising the Locus-2 *tctA4*/*tctB* pair, the orphan Bug-family substrate-binding protein *tctC4* and the Locus-1 *tctB*, layered on top of the *tph*-encoded *tctC* substrate-binding protein that is induced as part of the shared PET response; consistent with a putative role in TPA capture at the polymer interface, which was not tested functionally here. The single-cell Raman phenotype (protein-rich, saturated membrane; elevated cytochrome c) provides an independent read-out.

**Table S1. NCBI coordinate annotation of the 19 PET-catabolic pathway and biofilm-specific transporter genes.** For each gene: assigned module, gene symbol, NCBI RefSeq locus tag (assembly CP166677) and product, and the log_2_ fold change with Benjamini–Hochberg-adjusted P value in each of the three DESeq2 contrasts (biofilm-vs-maltose, BvM; planktonic-PET-vs-maltose, PvM; biofilm-vs-planktonic-on-PET, BvP). Prodigal gene identifiers and the complete module-wide statistics are in Table S2. n.s., padj not defined by DESeq2 independent filtering.

| **Module** | **Gene** | **NCBI locus tag** | **NCBI product** | **BvM log_2_FC (padj)** | **PvM log_2_FC (padj)** | **BvP log_2_FC (padj)** |
| --- | --- | --- | --- | --- | --- | --- |
| PET hydrolase | PETase | ABFT21_17780 | poly(ethylene terephthalate) hydrolase | +1.9 (1.7e-02) | +2.4 (5.7e-03) | -0.5 (7.2e-01) |
| PET hydrolase | MHETase | ABFT21_17715 | mono(2-hydroxyethyl) terephthalate hydrolase | +4.2 (2.2e-10) | +2.1 (1.8e-03) | +2.1 (n.s.) |
| TPA ring catabolism | *tctC (TPA)* | ABFT21_17730 | Bug family tripartite tricarboxylate transporter substrate binding protein | +5.0 (7.4e-12) | +3.4 (3.8e-07) | +1.6 (2.4e-01) |
| TPA ring catabolism | *tphA2* | ABFT21_17735 | aromatic ring-hydroxylating dioxygenase subunit alpha | +4.8 (1.1e-11) | +3.5 (6.9e-07) | +1.3 (3.5e-01) |
| TPA ring catabolism | *tphA3* | ABFT21_17740 | nuclear transport factor 2 family protein | +4.6 (2.1e-10) | +3.3 (2.9e-06) | +1.3 (3.3e-01) |
| TPA ring catabolism | *tphA1* | ABFT21_17750 | 2Fe-2S iron-sulfur cluster-binding protein | +3.5 (7.4e-07) | +2.8 (7.1e-05) | +0.6 (6.0e-01) |
| TPA ring catabolism | *tphB* | ABFT21_17745 | PdxA family protein | +4.2 (1.6e-08) | +3.4 (1.3e-06) | +0.7 (5.7e-01) |
| PCA ortho-cleavage | *pcaG* | ABFT21_01110 | protocatechuate 3,4-dioxygenase subunit alpha | +1.8 (5.3e-05) | +0.9 (8.5e-02) | +0.9 (1.7e-01) |
| PCA ortho-cleavage | *pcaB2* | ABFT21_23295 | adenylosuccinate lyase family protein | +1.5 (4.3e-06) | +1.5 (3.9e-03) | -0.0 (9.6e-01) |
| PCA ortho-cleavage | *pcaD1* | ABFT21_03945 | alpha/beta fold hydrolase | +2.0 (1.4e-03) | +1.4 (8.7e-02) | +0.6 (4.2e-01) |
| PCA ortho-cleavage | *pcaD3* | ABFT21_16325 | alpha/beta fold hydrolase | +1.2 (1.6e-03) | +1.0 (4.5e-02) | +0.2 (6.6e-01) |
| PCA ortho-cleavage | *pcaI4* | ABFT21_15095 | 3-oxoacid CoA-transferase subunit A | +1.6 (2.5e-04) | +1.3 (2.9e-03) | +0.4 (5.9e-01) |
| EG pathway | *pedE* | ABFT21_21420 | methanol/ethanol family PQQ-dependent dehydrogenase | +1.5 (4.5e-03) | +0.8 (4.4e-02) | +0.6 (4.1e-01) |
| EG pathway | *xoxF* | ABFT21_19395 | methanol/ethanol family PQQ-dependent dehydrogenase | +0.1 (7.6e-01) | +1.4 (1.7e-02) | -1.3 (5.9e-02) |
| EG pathway | *glcD* | ABFT21_01215 | FAD-linked oxidase C-terminal domain-containing protein | -1.4 (3.8e-05) | -0.5 (4.2e-01) | -1.0 (1.9e-01) |
| Biofilm-specific TTT transport | *tctA (loc.2)* | ABFT21_18265 | tripartite tricarboxylate transporter permease | +1.8 (5.8e-03) | -0.6 (2.3e-01) | +2.5 (1.6e-03) |
| Biofilm-specific TTT transport | *tctB (loc.2)* | ABFT21_18270 | tripartite tricarboxylate transporter TctB family protein | +1.8 (5.9e-03) | -1.3 (3.2e-02) | +3.1 (4.9e-04) |
| Biofilm-specific TTT transport | *tctC4 (orphan)* | ABFT21_05640 | tripartite tricarboxylate transporter substrate binding protein BugD | +2.6 (1.4e-04) | -0.3 (6.7e-01) | +2.9 (5.3e-03) |
| Biofilm-specific TTT transport | *tctB (loc.1)* | ABFT21_04760 | tripartite tricarboxylate transporter TctB family protein | +1.9 (7.4e-04) | +0.2 (7.8e-01) | +1.8 (1.3e-02) |

**Table S2. Differential-expression statistics for the 139 genes of the coordinated PET-response modules.** log_2_ fold change and Benjamini–Hochberg-adjusted P value for every gene of the PET-catabolic pathway and of the flagellar/chemotaxis, TCA-cycle, exopolysaccharide/matrix-biosynthesis and membrane-lipid-remodelling lifestyle modules (Fig. 3c and Fig. S5), across the three DESeq2 contrasts (BvM, PvM, BvP).

| **Module** | **Gene** | **NCBI locus tag** | **NCBI product** | **BvM log_2_FC (padj)** | **PvM log_2_FC (padj)** | **BvP log_2_FC (padj)** |
| --- | --- | --- | --- | --- | --- | --- |
| PET pathway: PET hydrolase | PETase | ABFT21_17780 | poly(ethylene terephthalate) hydrolase | +1.9 (1.7e-02) | +2.4 (5.7e-03) | -0.5 (7.2e-01) |
| PET pathway: PET hydrolase | MHETase | ABFT21_17715 | mono(2-hydroxyethyl) terephthalate hydrolase | +4.2 (2.2e-10) | +2.1 (1.8e-03) | +2.1 (n.s.) |
| PET pathway: TPA ring catabolism | tctC (TPA-binding) | ABFT21_17730 | Bug family tripartite tricarboxylate transportersubstrate binding protein | +5.0 (7.4e-12) | +3.4 (3.8e-07) | +1.6 (2.4e-01) |
| PET pathway: TPA ring catabolism | *tphA2* | ABFT21_17735 | aromatic ring-hydroxylating dioxygenase subunitalpha | +4.8 (1.1e-11) | +3.5 (6.9e-07) | +1.3 (3.5e-01) |
| PET pathway: TPA ring catabolism | *tphA3* | ABFT21_17740 | nuclear transport factor 2 family protein | +4.6 (2.1e-10) | +3.3 (2.9e-06) | +1.3 (3.3e-01) |
| PET pathway: TPA ring catabolism | *tphA1* | ABFT21_17750 | 2Fe-2S iron-sulfur cluster-binding protein | +3.5 (7.4e-07) | +2.8 (7.1e-05) | +0.6 (6.0e-01) |
| PET pathway: TPA ring catabolism | *tphB* | ABFT21_17745 | PdxA family protein | +4.2 (1.6e-08) | +3.4 (1.3e-06) | +0.7 (5.7e-01) |
| PET pathway: PCA ortho- cleavage | *pcaG* | ABFT21_01110 | protocatechuate 3,4-dioxygenase subunit alpha | +1.8 (5.3e-05) | +0.9 (8.5e-02) | +0.9 (1.7e-01) |
| PET pathway: PCA ortho- cleavage | *pcaB2* | ABFT21_23295 | adenylosuccinate lyase family protein | +1.5 (4.3e-06) | +1.5 (3.9e-03) | -0.0 (9.6e-01) |
| PET pathway: PCA ortho- cleavage | *pcaD1* | ABFT21_03945 | alpha/beta fold hydrolase | +2.0 (1.4e-03) | +1.4 (8.7e-02) | +0.6 (4.2e-01) |
| PET pathway: PCA ortho- cleavage | *pcaD3* | ABFT21_16325 | alpha/beta fold hydrolase | +1.2 (1.6e-03) | +1.0 (4.5e-02) | +0.2 (6.6e-01) |
| PET pathway: PCA ortho- cleavage | *pcaI4* | ABFT21_15095 | 3-oxoacid CoA-transferase subunit A | +1.6 (2.5e-04) | +1.3 (2.9e-03) | +0.4 (5.9e-01) |
| PET pathway: EG pathway | *pedE* | ABFT21_21420 | methanol/ethanol family PQQ-dependentdehydrogenase | +1.5 (4.5e-03) | +0.8 (4.4e-02) | +0.6 (4.1e-01) |
| PET pathway: EG pathway | *xoxF* | ABFT21_19395 | methanol/ethanol family PQQ-dependentdehydrogenase | +0.1 (7.6e-01) | +1.4 (1.7e-02) | -1.3 (5.9e-02) |
| PET pathway: EG pathway | *glcD* | ABFT21_01215 | FAD-linked oxidase C-terminal domain-containingprotein | -1.4 (3.8e-05) | -0.5 (4.2e-01) | -1.0 (1.9e-01) |
| PET pathway: Biofilm-specific TTT transport | tctA (loc.2) | ABFT21_18265 | tripartite tricarboxylate transporter permease | +1.8 (5.8e-03) | -0.6 (2.3e-01) | +2.5 (1.6e-03) |
| PET pathway: Biofilm-specific TTT transport | tctB (loc.2) | ABFT21_18270 | tripartite tricarboxylate transporter TctBfamily protein | +1.8 (5.9e-03) | -1.3 (3.2e-02) | +3.1 (4.9e-04) |
| PET pathway: Biofilm-specific TTT transport | tctC4 (orphan) | ABFT21_05640 | tripartite tricarboxylate transporter substratebinding protein BugD | +2.6 (1.4e-04) | -0.3 (6.7e-01) | +2.9 (5.3e-03) |
| PET pathway: Biofilm-specific TTT transport | tctB (loc.1) | ABFT21_04760 | tripartite tricarboxylate transporter TctBfamily protein | +1.9 (7.4e-04) | +0.2 (7.8e-01) | +1.8 (1.3e-02) |
| Flagellar & chemotaxis |  | ABFT21_01500 | methyl-accepting chemotaxis protein | -0.8 (5.4e-03) | -1.0 (4.9e-03) | +0.2 (6.9e-01) |
| Flagellar & chemotaxis |  | ABFT21_02035 | methyl-accepting chemotaxis protein | +1.6 (5.3e-03) | +1.0 (2.0e-01) | +0.5 (4.6e-01) |
| Flagellar & chemotaxis |  | ABFT21_02490 | methyl-accepting chemotaxis protein | +2.1 (2.9e-04) | +1.6 (4.7e-02) | +0.5 (5.4e-01) |
| Flagellar & chemotaxis |  | ABFT21_02735 | chemotaxis protein CheA | -0.6 (2.0e-02) | -0.9 (2.4e-02) | +0.3 (4.1e-01) |
| Flagellar & chemotaxis |  | ABFT21_02740 | methyl-accepting chemotaxis protein | -2.0 (8.5e-09) | -1.9 (6.1e-09) | -0.1 (8.1e-01) |
| Flagellar & chemotaxis |  | ABFT21_02745 | chemotaxis protein CheW | -0.8 (7.6e-03) | -1.2 (1.1e-02) | +0.4 (4.0e-01) |
| Flagellar & chemotaxis | *flgM* | ABFT21_06535 | flagellar biosynthesis anti-sigma factor FlgM | -3.9 (1.4e-16) | -2.8 (7.2e-06) | -1.2 (2.2e-01) |
| Flagellar & chemotaxis | *flgA* | ABFT21_06540 | flagellar basal body P-ring formation chaperoneFlgA | -1.3 (6.1e-05) | -1.2 (2.8e-03) | -0.2 (6.4e-01) |
| Flagellar & chemotaxis |  | ABFT21_06545 | flagellar basal body rod protein FlgB | -1.8 (1.8e-07) | -1.2 (1.1e-02) | -0.7 (1.2e-01) |
| Flagellar & chemotaxis | *flgC* | ABFT21_06550 | flagellar basal body rod protein FlgC | -1.7 (2.6e-03) | -1.3 (n.s.) | -0.4 (3.0e-01) |
| Flagellar & chemotaxis |  | ABFT21_06555 | flagellar hook assembly protein FlgD | -2.0 (5.6e-06) | -1.2 (2.9e-02) | -0.8 (3.8e-02) |
| Flagellar & chemotaxis | *flgE* | ABFT21_06560 | flagellar hook protein FlgE | -2.6 (1.1e-07) | -1.7 (5.8e-03) | -1.0 (1.1e-01) |
| Flagellar & chemotaxis | *flgF* | ABFT21_06565 | flagellar basal-body rod protein FlgF | -1.7 (4.0e-04) | -1.4 (1.1e-02) | -0.3 (3.5e-01) |
| Flagellar & chemotaxis | *flgG* | ABFT21_06570 | flagellar basal-body rod protein FlgG | -1.5 (6.6e-04) | -1.1 (4.9e-02) | -0.5 (2.2e-01) |
| Flagellar & chemotaxis |  | ABFT21_06575 | flagellar basal body L-ring protein FlgH | -1.9 (3.7e-06) | +0.1 (8.9e-01) | -2.1 (1.2e-02) |
| Flagellar & chemotaxis |  | ABFT21_06580 | flagellar basal body P-ring protein FlgI | -0.9 (2.6e-02) | -0.7 (1.6e-01) | -0.3 (2.5e-01) |
| Flagellar & chemotaxis | *flgL* | ABFT21_06595 | flagellar hook-associated protein FlgL | -0.8 (3.3e-02) | -0.4 (4.0e-01) | -0.4 (3.9e-02) |
| Flagellar & chemotaxis |  | ABFT21_08220 | methyl-accepting chemotaxis protein | -3.1 (1.5e-21) | -2.4 (3.0e-08) | -0.7 (1.2e-01) |
| Flagellar & chemotaxis |  | ABFT21_08225 | chemotaxis protein CheW | -4.3 (2.5e-21) | -2.6 (3.9e-06) | -1.8 (8.5e-04) |
| Flagellar & chemotaxis |  | ABFT21_08230 | methyl-accepting chemotaxis protein | -2.4 (9.9e-19) | -2.0 (8.3e-11) | -0.4 (1.2e-01) |
| Flagellar & chemotaxis |  | ABFT21_08235 | chemotaxis protein CheA | -2.1 (6.5e-11) | -1.8 (9.4e-09) | -0.3 (2.3e-01) |
| Flagellar & chemotaxis |  | ABFT21_08545 | chemotaxis response regulator protein-glutamatemethylesterase | -1.0 (5.1e-03) | -1.3 (3.4e-03) | +0.3 (5.3e-01) |
| Flagellar & chemotaxis |  | ABFT21_11050 | methyl-accepting chemotaxis protein | -1.6 (4.9e-08) | -1.7 (2.9e-07) | +0.1 (8.5e-01) |
| Flagellar & chemotaxis |  | ABFT21_12180 | chemotaxis protein CheW | +1.2 (2.5e-04) | +0.8 (8.5e-02) | +0.3 (4.1e-01) |
| Flagellar & chemotaxis |  | ABFT21_12785 | methyl-accepting chemotaxis protein | +0.5 (3.2e-03) | +0.4 (1.3e-01) | -0.0 (9.7e-01) |
| Flagellar & chemotaxis |  | ABFT21_13155 | methyl-accepting chemotaxis protein | -0.5 (2.4e-02) | -0.3 (3.7e-01) | -0.3 (1.2e-01) |
| Flagellar & chemotaxis | *flhF* | ABFT21_13425 | flagellar biosynthesis protein FlhF | -0.9 (8.7e-03) | -0.7 (1.0e-01) | -0.2 (4.7e-01) |
| Flagellar & chemotaxis | *cheY* | ABFT21_14880 | chemotaxis response regulator CheY | -4.0 (2.9e-25) | -3.0 (4.1e-12) | -1.0 (9.9e-02) |
| Flagellar & chemotaxis | *motB* | ABFT21_14885 | flagellar motor protein MotB | -2.6 (2.7e-15) | -1.9 (1.1e-06) | -0.8 (2.3e-02) |
| Flagellar & chemotaxis | *motA* | ABFT21_14890 | flagellar motor stator protein MotA | -2.8 (3.6e-29) | -1.8 (7.8e-07) | -1.1 (1.3e-02) |
| Flagellar & chemotaxis | *flhC* | ABFT21_14895 | flagellar transcriptional regulator FlhC | -2.4 (1.1e-04) | -1.3 (7.6e-02) | -1.1 (1.5e-01) |
| Flagellar & chemotaxis | *flhD* | ABFT21_14900 | flagellar transcriptional regulator FlhD | -3.0 (5.0e-07) | -1.6 (3.8e-02) | -1.4 (9.5e-02) |
| Flagellar & chemotaxis |  | ABFT21_14970 | flagellin domain-containing protein | -5.2 (1.4e-41) | -3.3 (4.4e-08) | -2.0 (6.3e-03) |
| Flagellar & chemotaxis |  | ABFT21_14975 | flagellin | -2.8 (4.6e-08) | -1.5 (2.7e-02) | -1.4 (9.9e-02) |
| Flagellar & chemotaxis | *fliD* | ABFT21_14980 | flagellar filament capping protein FliD | -1.7 (1.0e-06) | -1.3 (2.8e-04) | -0.4 (8.1e-02) |
| Flagellar & chemotaxis | *fliS* | ABFT21_14985 | flagellar export chaperone FliS | -3.1 (4.3e-12) | -2.3 (1.6e-06) | -0.9 (7.3e-02) |
| Flagellar & chemotaxis |  | ABFT21_14990 | flagellar protein FliT | -3.4 (1.7e-17) | -2.8 (1.9e-12) | -0.6 (2.3e-01) |
| Flagellar & chemotaxis |  | ABFT21_14995 | flagellar brake protein | -1.4 (1.2e-04) | -1.1 (1.5e-02) | -0.4 (2.2e-01) |
| Flagellar & chemotaxis | *fliE* | ABFT21_15000 | flagellar hook-basal body complex protein FliE | -0.6 (2.5e-02) | -0.3 (3.4e-01) | -0.3 (2.5e-01) |
| Flagellar & chemotaxis | *fliF* | ABFT21_15005 | flagellar basal-body MS-ring/collar proteinFliF | -1.0 (4.3e-03) | -0.6 (1.7e-01) | -0.5 (3.8e-02) |
| Flagellar & chemotaxis | *fliG* | ABFT21_15010 | flagellar motor switch protein FliG | -2.0 (3.2e-06) | -1.0 (4.7e-02) | -1.0 (2.4e-02) |
| Flagellar & chemotaxis | *fliL* | ABFT21_15035 | flagellar basal body-associated protein FliL | -1.8 (3.3e-07) | -1.7 (1.3e-04) | -0.2 (6.5e-01) |
| Flagellar & chemotaxis | *fliM* | ABFT21_15040 | flagellar motor switch protein FliM | -2.1 (5.1e-07) | -1.4 (2.1e-02) | -0.7 (2.2e-01) |
| Flagellar & chemotaxis | *fliN* | ABFT21_15045 | flagellar motor switch protein FliN | -1.4 (2.3e-04) | -1.3 (1.8e-02) | -0.1 (8.3e-01) |
| Flagellar & chemotaxis |  | ABFT21_15775 | methyl-accepting chemotaxis protein | -2.0 (2.2e-10) | -1.4 (1.3e-03) | -0.7 (1.9e-01) |
| Flagellar & chemotaxis |  | ABFT21_16315 | methyl-accepting chemotaxis protein | -1.9 (3.6e-12) | -1.1 (1.3e-02) | -0.8 (1.1e-01) |
| Flagellar & chemotaxis |  | ABFT21_17505 | methyl-accepting chemotaxis protein | -4.0 (1.4e-25) | -3.1 (2.7e-14) | -0.9 (4.8e-03) |
| Flagellar & chemotaxis |  | ABFT21_18780 | methyl-accepting chemotaxis protein | +1.4 (1.1e-03) | +0.9 (1.9e-01) | +0.5 (4.3e-01) |
| Flagellar & chemotaxis |  | ABFT21_20175 | methyl-accepting chemotaxis protein | -1.5 (4.3e-06) | -1.6 (1.1e-08) | +0.1 (7.2e-01) |
| Flagellar & chemotaxis |  | ABFT21_20275 | chemotaxis protein CheB | +0.9 (1.4e-04) | +0.8 (1.9e-02) | +0.1 (6.7e-01) |
| Flagellar & chemotaxis |  | ABFT21_21495 | methyl-accepting chemotaxis protein | -2.6 (5.5e-15) | -2.2 (3.8e-09) | -0.4 (3.0e-01) |
| TCA cycle |  | ABFT21_10565 | fumarate hydratase | -1.5 (1.4e-05) | -1.3 (6.5e-04) | -0.2 (5.2e-01) |
| TCA cycle |  | ABFT21_11265 | 2-oxoglutarate dehydrogenase E1 component | -2.5 (6.3e-07) | -2.5 (1.8e-08) | -0.0 (9.7e-01) |
| TCA cycle | *odhB* | ABFT21_11270 | 2-oxoglutarate dehydrogenase complexdihydrolipoyllysine-residue succinyltransferase | -2.5 (7.2e-15) | -2.7 (2.9e-17) | +0.1 (7.3e-01) |
| TCA cycle | *icd* | ABFT21_17865 | NADP-dependent isocitrate dehydrogenase | -2.7 (2.8e-05) | -2.3 (4.9e-04) | -0.5 (7.1e-01) |
| TCA cycle | *leuB* | ABFT21_20050 | 3-isopropylmalate dehydrogenase | -1.5 (4.0e-05) | -1.1 (3.8e-03) | -0.4 (3.8e-01) |
| TCA cycle |  | ABFT21_20080 | citrate synthase | -1.4 (2.2e-03) | -1.2 (1.6e-02) | -0.2 (7.3e-01) |
| TCA cycle |  | ABFT21_20090 | succinate dehydrogenase iron-sulfur subunit | -2.0 (2.0e-05) | -1.6 (1.8e-03) | -0.4 (6.0e-01) |
| TCA cycle | *sdhA* | ABFT21_20095 | succinate dehydrogenase flavoprotein subunit | -1.8 (1.5e-05) | -1.4 (1.9e-03) | -0.4 (5.3e-01) |
| TCA cycle | *sdhD* | ABFT21_20100 | succinate dehydrogenase, hydrophobic membraneanchor protein | -2.0 (2.5e-04) | -1.2 (4.4e-02) | -0.7 (4.5e-01) |
| TCA cycle |  | ABFT21_20115 | malate dehydrogenase | -2.4 (1.4e-07) | -2.2 (9.0e-07) | -0.3 (7.4e-01) |
| TCA cycle | *tam* | ABFT21_20120 | trans-aconitate 2-methyltransferase | -0.7 (2.5e-02) | -1.4 (2.2e-03) | +0.6 (6.9e-02) |
| TCA cycle | *acnB* | ABFT21_20145 | bifunctional aconitate hydratase2/2-methylisocitrate dehydratase | -2.2 (3.8e-08) | -1.9 (6.7e-07) | -0.3 (5.7e-01) |
| TCA cycle |  | ABFT21_20160 | aconitate hydratase | -1.3 (5.1e-05) | -0.7 (1.4e-01) | -0.7 (2.3e-01) |
| TCA cycle |  | ABFT21_23680 | succinate dehydrogenase/fumarate reductaseiron-sulfur subunit | +2.1 (2.8e-03) | +1.7 (6.4e-02) | +0.4 (5.5e-01) |
| TCA cycle |  | ABFT21_23685 | succinate dehydrogenase | +2.3 (3.9e-03) | +1.7 (1.5e-01) | +0.6 (4.0e-01) |
| TCA cycle |  | ABFT21_23705 | fumarate hydratase C-terminal domain-containingprotein | +1.9 (2.3e-02) | +1.4 (2.7e-01) | +0.5 (5.8e-01) |
| TCA cycle | *aceK* | ABFT21_25760 | bifunctional isocitrate dehydrogenasekinase/phosphatase | -1.2 (1.8e-04) | -0.9 (2.2e-02) | -0.3 (4.6e-01) |
| Exopolysaccharide / matrix | *waaF* | ABFT21_02375 | lipopolysaccharide heptosyltransferase II | +0.8 (3.3e-02) | +0.2 (7.1e-01) | +0.5 (1.8e-01) |
| Exopolysaccharide / matrix |  | ABFT21_02940 | MSMEG_0565 family glycosyltransferase | +2.3 (4.8e-02) | +1.7 (3.5e-01) | +0.6 (4.1e-01) |
| Exopolysaccharide / matrix |  | ABFT21_03420 | glycosyltransferase family 4 protein | +1.9 (1.3e-02) | +1.4 (1.5e-01) | +0.5 (4.6e-01) |
| Exopolysaccharide / matrix |  | ABFT21_03855 | TIGR04282 family arsenosugar biosynthesisglycosyltransferase | +1.8 (4.9e-11) | +1.3 (5.7e-04) | +0.4 (8.2e-02) |
| Exopolysaccharide / matrix |  | ABFT21_03860 | TIGR04283 family arsenosugar biosynthesisglycosyltransferase | +1.2 (2.1e-04) | +0.9 (1.0e-02) | +0.2 (4.9e-01) |
| Exopolysaccharide / matrix |  | ABFT21_04575 | glycosyltransferase | +2.1 (1.5e-02) | +1.3 (2.1e-01) | +0.7 (2.0e-01) |
| Exopolysaccharide / matrix |  | ABFT21_07020 | glycosyltransferase | +1.0 (2.0e-02) | +0.5 (4.3e-01) | +0.5 (4.4e-01) |
| Exopolysaccharide / matrix |  | ABFT21_07040 | glycosyltransferase | +1.6 (3.2e-04) | +1.1 (8.6e-02) | +0.5 (2.8e-01) |
| Exopolysaccharide / matrix |  | ABFT21_07045 | glycosyltransferase family 2 protein | +1.8 (4.1e-05) | +1.3 (1.8e-02) | +0.4 (3.7e-01) |
| Exopolysaccharide / matrix |  | ABFT21_07065 | polysaccharide biosynthesis protein | +1.4 (7.1e-04) | +1.0 (1.4e-01) | +0.4 (5.5e-01) |
| Exopolysaccharide / matrix |  | ABFT21_07145 | glycosyltransferase family 9 protein | +1.1 (2.3e-02) | +0.6 (4.2e-01) | +0.5 (4.7e-01) |
| Exopolysaccharide / matrix |  | ABFT21_07155 | glycosyltransferase | +1.1 (8.1e-03) | +0.7 (1.9e-01) | +0.3 (4.7e-01) |
| Exopolysaccharide / matrix |  | ABFT21_07160 | glycosyltransferase family 4 protein | +0.7 (3.2e-02) | +0.3 (4.8e-01) | +0.3 (3.3e-01) |
| Exopolysaccharide / matrix |  | ABFT21_07190 | lipopolysaccharide biosynthesis protein | +1.6 (2.1e-02) | +1.2 (1.4e-01) | +0.3 (5.1e-01) |
| Exopolysaccharide / matrix |  | ABFT21_11055 | glycosyltransferase | +1.8 (4.6e-04) | +1.1 (8.7e-02) | +0.6 (3.0e-01) |
| Exopolysaccharide / matrix |  | ABFT21_15670 | O-antigen ligase family protein | -0.6 (8.6e-04) | -0.7 (1.4e-02) | +0.0 (9.1e-01) |
| Exopolysaccharide / matrix |  | ABFT21_15700 | glycosyltransferase family 2 protein | -1.1 (6.5e-04) | -0.9 (6.9e-02) | -0.3 (6.2e-01) |
| Exopolysaccharide / matrix |  | ABFT21_15705 | glycosyltransferase family 2 protein | -1.0 (2.7e-02) | -0.5 (3.6e-01) | -0.4 (3.3e-01) |
| Exopolysaccharide / matrix |  | ABFT21_15735 | O-antigen ligase family protein | +1.2 (8.0e-04) | +1.0 (4.5e-02) | +0.2 (6.9e-01) |
| Exopolysaccharide / matrix |  | ABFT21_15745 | WcaI family glycosyltransferase | +2.2 (5.8e-05) | +1.8 (2.0e-02) | +0.3 (6.4e-01) |
| Exopolysaccharide / matrix |  | ABFT21_17830 | glycosyltransferase | -0.8 (6.1e-04) | +0.0 (9.4e-01) | -0.9 (7.3e-02) |
| Exopolysaccharide / matrix |  | ABFT21_20460 | glycosyltransferase family 2 protein | -2.2 (2.7e-05) | -0.7 (3.6e-01) | -1.5 (1.7e-01) |
| Exopolysaccharide / matrix |  | ABFT21_20475 | glycosyltransferase family 2 protein | +0.9 (1.3e-02) | +0.6 (3.0e-01) | +0.2 (6.6e-01) |
| Exopolysaccharide / matrix |  | ABFT21_20485 | glycosyltransferase family 2 protein | +2.3 (1.1e-03) | +1.6 (6.3e-02) | +0.6 (2.2e-01) |
| Exopolysaccharide / matrix | *waaC* | ABFT21_20875 | lipopolysaccharide heptosyltransferase I | +1.0 (2.5e-02) | +0.1 (8.9e-01) | +0.9 (2.0e-01) |
| Exopolysaccharide / matrix | *lptA* | ABFT21_24010 | lipopolysaccharide transport periplasmic proteinLptA | -1.6 (1.6e-04) | -0.5 (4.5e-01) | -1.2 (1.7e-01) |
| Exopolysaccharide / matrix |  | ABFT21_24505 | glycosyltransferase | +0.9 (6.6e-03) | +1.0 (4.7e-02) | -0.1 (8.8e-01) |
| Exopolysaccharide / matrix |  | ABFT21_24550 | polysaccharide biosynthesis/export familyprotein | -1.5 (1.2e-05) | -1.2 (1.1e-03) | -0.3 (6.1e-01) |
| Exopolysaccharide / matrix |  | ABFT21_24555 | glycosyltransferase | -0.7 (1.6e-02) | -0.6 (1.8e-01) | -0.1 (7.2e-01) |
| Exopolysaccharide / matrix |  | ABFT21_24560 | glycosyltransferase | -0.8 (2.0e-02) | -0.5 (2.0e-01) | -0.3 (4.2e-01) |
| Exopolysaccharide / matrix |  | ABFT21_24715 | glycosyltransferase | -0.5 (4.0e-03) | -0.8 (1.0e-02) | +0.3 (3.3e-01) |
| Exopolysaccharide / matrix |  | ABFT21_24720 | glycosyltransferase | -0.6 (7.6e-03) | -1.0 (2.9e-03) | +0.3 (1.7e-01) |
| Exopolysaccharide / matrix |  | ABFT21_24755 | putative capsular polysaccharide synthesisfamily protein | -1.8 (1.4e-04) | -0.7 (2.6e-01) | -1.1 (1.9e-01) |
| Exopolysaccharide / matrix |  | ABFT21_24805 | glycosyltransferase | -2.6 (3.6e-07) | -2.0 (2.3e-03) | -0.6 (5.5e-01) |
| Exopolysaccharide / matrix |  | ABFT21_24820 | glycosyltransferase | +0.6 (9.9e-04) | +0.3 (3.1e-01) | +0.3 (4.5e-02) |
| Exopolysaccharide / matrix |  | ABFT21_24840 | glycosyltransferase family 4 protein | -0.9 (6.7e-05) | -0.7 (1.6e-02) | -0.2 (4.6e-01) |
| Exopolysaccharide / matrix |  | ABFT21_25540 | lipopolysaccharide biosynthesis protein | +1.3 (4.0e-02) | +0.7 (4.4e-01) | +0.6 (3.3e-01) |
| Exopolysaccharide / matrix |  | ABFT21_25550 | glycosyltransferase family 4 protein | +1.4 (2.8e-03) | +0.7 (2.6e-01) | +0.6 (2.2e-01) |
| Exopolysaccharide / matrix |  | ABFT21_25570 | glycosyltransferase family 2 protein | +1.0 (5.6e-05) | +0.7 (1.9e-01) | +0.2 (6.5e-01) |
| Exopolysaccharide / matrix |  | ABFT21_25590 | lipopolysaccharide biosynthesis protein | +1.1 (5.5e-03) | +0.7 (1.5e-01) | +0.3 (1.4e-01) |
| Membrane-lipid remodelling | *cfa* | ABFT21_01130 | cyclopropane fatty acyl phospholipid synthase | +1.6 (3.9e-07) | +1.6 (8.4e-06) | -0.0 (9.0e-01) |
| Membrane-lipid remodelling |  | ABFT21_04230 | lipid A biosynthesis acyltransferase | +1.0 (8.4e-03) | +0.6 (2.3e-01) | +0.3 (6.2e-01) |
| Membrane-lipid remodelling |  | ABFT21_06740 | beta-ketoacyl synthase N-terminal-likedomain-containing protein | +0.5 (6.5e-02) | -0.1 (8.9e-01) | +0.5 (9.6e-03) |
| Membrane-lipid remodelling |  | ABFT21_06755 | lysophospholipid acyltransferase family protein | +0.8 (1.4e-03) | +0.2 (7.7e-01) | +0.5 (3.3e-01) |
| Membrane-lipid remodelling |  | ABFT21_06765 | beta-ketoacyl synthase chain length factor | +1.3 (7.5e-04) | +0.8 (9.2e-02) | +0.5 (1.4e-01) |
| Membrane-lipid remodelling |  | ABFT21_06770 | beta-ketoacyl synthase chain length factor | +0.8 (1.2e-02) | +0.4 (3.5e-01) | +0.4 (1.4e-01) |
| Membrane-lipid remodelling |  | ABFT21_06775 | beta-ketoacyl-[acyl-carrier-protein] synthasefamily protein | +0.7 (1.0e-02) | +0.3 (5.6e-01) | +0.4 (2.5e-01) |
| Membrane-lipid remodelling |  | ABFT21_07590 | lysylphosphatidylglycerol synthasedomain-containing protein | +2.3 (4.9e-02) | +1.7 (1.8e-01) | +0.5 (2.6e-01) |
| Membrane-lipid remodelling | *fabF* | ABFT21_18505 | beta-ketoacyl-ACP synthase II | -2.1 (4.0e-06) | -2.0 (1.2e-05) | -0.1 (8.6e-01) |
| Membrane-lipid remodelling |  | ABFT21_18525 | beta-ketoacyl-ACP synthase III | -1.2 (2.8e-03) | -1.3 (5.2e-03) | +0.1 (9.0e-01) |
| Membrane-lipid remodelling |  | ABFT21_21400 | lysylphosphatidylglycerol synthasedomain-containing protein | +2.8 (2.9e-03) | +2.4 (2.4e-02) | +0.4 (2.2e-01) |
| Membrane-lipid remodelling |  | ABFT21_21835 | lysophospholipid acyltransferase family protein | +1.6 (8.9e-05) | +1.0 (1.3e-01) | +0.6 (3.2e-01) |

**Table S3.** Quantitative confocal live/dead read-outs for the four amino-acid supplementation conditions.

| **Condition** | **SYTO9^+^ area coverage (% FOV)^a^** | **Viable surface fraction (%)^b^** |
| --- | --- | --- |
| SV control | 2.8 | 97.5 |
| +Val | 1.8 | 21.9 |
| +Ile | 14.7 | 99.9 |
| +Ser | 12.5 | 99.7 |

^a^ Fraction of maximum-intensity-projection pixels above an Otsu SYTO9 threshold (8-bit floor 60 digital number units). ^b^ N(SYTO9^+^ PI^–^) ÷ N(SYTO9^+^ PI^–^ + SYTO9^+^ PI^+^ + SYTO9^–^PI^+^) across Imaris-segmented surface objects, using fixed intensity thresholds SYTO9 > 30 and PI > 50 anchored to the SV-control bimodal split. Background-only surfaces excluded.

**Extended methods**

***Characterization of PET***

**Gravimetric weight loss.** After 4 weeks of incubation, coupons were retrieved and cleaned of adherent biomass using a two-step wash: the coupon was blotted on a Kimwipe and immersed in 2 % SDS, incubated at 30 °C for 30 min with shaking at 200 rpm, blotted again and immersed in 70 % ethanol, then incubated at 30 °C for 30 min with shaking at 200 rpm. Cleaned coupons were air-dried overnight and reweighed used pre-incubation. Weight loss is reported as the absolute mass difference (mg). The full design comprised *n* = 4 PET coupons per group (PET only: sterile no-bacteria control; PS + PET: *P. sakaiensis* inoculated).

**Scanning electron microscopy.** Samples were imaged using a JEOL JSM-7600F scanning electron microscope operated at an accelerating voltage of 2 kV in LABE GB-high mode, with a working distance of 6 mm. Prior to imaging, samples were sputter-coated. Images were acquired at 5000× magnification. A representative SEM micrograph is shown in Fig. 1a as part of the experimental workflow schematic (scale bar: 10 µm).

**Thermogravimetric analysis.** Recovered PET coupons analysed by TGA on a PerkinElmer Thermal Analyser 4000 at a constant heating rate of 10 °C/min from 30 °C to 700 °C under a N_2_ atmosphere. The 5 % and 10 % mass-loss onset temperatures (T_5_% and T_10_%) were extracted from each mass-loss curve, and Δ values reported as PS+PET − PET only.

**TG-FTIR evolved-gas analysis.** Evolved gases from the TGA furnace were routed in real time to a coupled PerkinElmer Fourier-transform infrared spectrometer; absorbance spectra of the gas stream were acquired across the full thermal ramp and processed in PerkinElmer Spectrum TimeBase. The spectrum at each sample’s peak gas-evolution temperature was extracted; difference (Δ Absorbance) is plotted against wavenumber. Per-band intensities were integrated at the canonical pyrolysis-product bands (H_2_O 3650, aliphatic C–H 2950, CO_2_ 2350, ester C=O 1745, aromatic ring 1600, C–O 1240 cm^-1^) and PS / PET fold changes computed. The companion Gram–Schmidt root-mean-square trace (total IR absorbance vs T) was integrated over the 380–500 °C primary decomposition window to give a temperature-resolved PS / PET evolved-gas ratio that confirms enhanced volatile release across the full window rather than at a single isolated temperature.

***RNA extraction, sequencing, and bioinformatics***

**Experimental design and sample collection.** Five biological replicates per condition across three conditions (Biofilm on PET [B], Planktonic on PET [P], Planktonic on maltose [M]) yielded 15 libraries total. Biofilm RNA was extracted from cells adherent to the PET surface; planktonic RNA from cells in suspension. RNA was stabilised in nuclease free water at -80 ^o^C immediately after elution.

**Extraction, library preparation, and sequencing.** Total RNA was extracted using the SPINeasy RNA Kit for Bacteria (with Lysing Matrix; MP Biomedicals), followed by DNase treatment using the RNase-Free DNase Set (Qiagen). RNA was subsequently purified and concentrated using the Zymo RNA Clean & Concentrator-25 kit to obtain high-quality total RNA. Ribosomal RNA was depleted prior to library construction.

Illumina sequencing libraries were prepared using the Swift RNA Library Kit with Swift Unique Dual Indexing Kit and sequenced on an Illumina HiSeq X platform (v2.5 chemistry; 150 × 150 bp paired-end reads). For Oxford Nanopore Technologies (ONT) sequencing, libraries were prepared using the SQK-NBD114 Native Barcoding Kit (V14) and sequenced on GridION/MinION devices using R10.4.1 flow cells.

**Reference genome and annotation.** The *P. sakaiensis* 201-F6 reference genome (NCBI accession CP166677, 5,572 predicted coding sequences) was used throughout. Protein-coding genes were predicted *de novo* with Prodigal and cross-referenced against PGAP and Prokka annotations; functional categories were assigned with eggNOG-mapper (COG categories) and KEGG KOALA (KO / pathway IDs). Tripartite tricarboxylate transporter (TTT) family genes were enumerated from eggNOG-mapper KEGG orthology assignments (K07795 *tctA*, K07796 *tctB*, K07797 *tctC*). For the pathway-level analysis reported in Fig. 3, Fig. S4, Fig. S5 and Tables S1 and S2, all 5,572 predicted coding sequences were additionally re-annotated by genome coordinate against the NCBI RefSeq annotation of the same assembly (CP166677): each Prodigal call was matched to the RefSeq CDS with which it overlapped, and the RefSeq locus tag, gene symbol and product were carried through. This step was necessary because several genes central to the analysis — including PETase, MHETase and the tripartite tricarboxylate transporter genes — fall in COG category S (“function unknown”) under the eggNOG annotation and are therefore invisible to category-level mining. Locus tags reported in the text and in Tables S1 and S2 are NCBI RefSeq tags.

**Read alignment, quantification, and differential expression.** Raw sequencing reads were quality-filtered using Trimmomatic (v0.39-2) to remove adapter sequences and low-quality bases, with trimming of the first 10 bp of each read. Read quality was assessed using FastQC (v0.12.1). Filtered reads were aligned to the *P. sakaiensis* assembled genome (BioProject PRJNA1112095; annotated using the NCBI Prokaryotic Genome Annotation Pipeline) using STAR aligner. Gene-level read counts were generated using featureCounts with strand-specific settings appropriate for the library preparation. Differential expression was analysed in DESeq2 under a three-contrast Wald-test design comparing biofilm on PET against planktonic on maltose (BvM), planktonic on PET against planktonic on maltose (PvM) and biofilm on PET against planktonic on PET (BvP). P values were adjusted by the Benjamini–Hochberg procedure; genes with padj < 0.05 and |log_2_FC| > 1.5 were considered differentially expressed.

***Raman data preprocessing and analysis***

**Preprocessing.** Spectra were baseline-corrected with asymmetric least squares (ALS) using λ = 10^4^ and p = 10^-3^ over ~10–20 iterations, smoothed by a Savitzky–Golay filter (window = 9 points, polynomial order = 3), and normalised by extended multiplicative signal correction (EMSC).

**Raman analysis.** EMSC-normalised spectra were reduced by principal component analysis and by t-distributed stochastic neighbour embedding (perplexity 30, seed 42). Supervised classification used linear discriminant analysis on the retained principal components with stratified 5-fold cross-validation, with per-fold PCA recomputed on the training split so that test-set scores never fed back into the training PCA.

Biomarker bands were quantified by trapezoidal band-area integration for broad bands and band-area ratios anchored at Cyt-*c* ν_15_ 750 cm^-1^, ν_19_ 1585 cm^-1^; Phe ring breath 1003 cm^-1^; lipid CH_2_ twist 1287 cm^-1^; CH deformation 1463 cm^-1^; CH_2_ symmetric stretch 2850 cm^-1^; =C-H *cis* stretch 3010 cm^-1^; CH_3_ asymmetric stretch 2930 cm^-1^; CH deformation 1450 cm^-1^; serine C–OH 876 cm^-1^; phosphatidylethanolamine head group 720 cm^-1^; polysaccharide 1080 cm^-1^; BCAA skeletal C–C 960 cm^-1^. Ratios were tested for group differences by two-sided Wilcoxon rank-sum tests. Both the per-wavenumber spectral tests (corrected within each contrast) and the summary band-area-ratio tests (corrected within each figure’s test family) were adjusted by the Benjamini–Hochberg procedure and are reported as q values.
